# Valorization of aquaculture side streams from sea bream and sea bass by enzymatic hydrolysis and fractionation: chemical and biological insights

**DOI:** 10.3389/fnut.2025.1663294

**Published:** 2025-09-26

**Authors:** Marte Jenssen, Izumi Sone, Federica Grasso, Federica Turrini, Francesca Tardanico, Giulia De Negri Atanasio, Diego Méndez Paz, Rebeca Vázquez Sobrado, Mercedes Alonso Martínez, Raffaella Boggia, Elena Grasselli, Kjersti Lian

**Affiliations:** ^1^Department of Marine Biotechnology, Norwegian Institute of Food, Fisheries and Aquaculture Research (Nofima), Tromsø, Norway; ^2^Department of Process Technology, Norwegian Institute of Food, Fisheries and Aquaculture Research (Nofima), Stavanger, Norway; ^3^Department of Pharmacy, University of Genova, Genova, Italy; ^4^National Center for the Development of New Technologies in Agriculture (Agritech), Napoli, Italy; ^5^Department of Earth, Environment and Life Science, University of Genoa, Genova, Italy; ^6^Interuniversity Center for the Promotion of 3R Principles in Teaching and Research (Centro 3R), Pisa, Italy; ^7^Department of Sustainability and Circular Economy, ANFACO-CECOPESCA, Vigo, Spain; ^8^Department of Biotechnology and Health, ANFACO-CECOPESCA, Vigo, Spain; ^9^National Biodiversity Future Center (NBFC), Palermo, Italy; ^10^Istituto Nazionale Biostrutture e Biosistemi (INBB), Rome, Italy

**Keywords:** enzymatic hydrolysis, fractionation, side stream utilization, aquaculture, sea bream, sea bass, bioactivity, techno-functional properties

## Abstract

Increased valorization of aquaculture side streams is essential for reducing waste and enhancing resource efficiency. This study investigates the enzymatic hydrolysis and subsequent fractionation of pre-processed side streams from sea bream and sea bass aquaculture. Using Corolase® 8000, hydrolysates were produced and fractionated into crude, permeate, and retentate using membrane filtration (3 kDa molecular weight cut-off). The samples were comprehensively characterized for chemical composition, techno-functional properties, and biological activities. Briefly, the chemical characterization revealed that all samples had high protein content (>80%). As expected, the highest average molecular weight was measured for the retentate, followed by the crude and the permeate. The permeate distinguishes itself from the other samples with a lighter color profile in the color analysis. In the techno-functional characterization, the retentate showed the most promising properties, surpassing in emulsifying activity, foaming capacity, and oil binding capacity, suggesting its potential in food applications. The crude had the highest stability to maintain foam and emulsion over time. The bioactivity screening revealed some activity in the hepatoprotective assay (reduced fatty acid accumulation), the cellular antioxidant assay, and the angiotensin-converting enzyme (ACE) inhibitory assay. The samples did not exhibit anti-inflammatory activity or anti-osteoporotic capacity. The most pronounced results from the bioactivity studies were found in the wound healing assay, where the permeate had significantly increased wound closure at all tested concentrations (0.05, 0.025, and 0.015 mg/mL), suggesting its potential for wound healing applications. These findings highlight the potential of fractionated fish protein hydrolysates as functional ingredients in nutraceutical and food formulations, supporting circular economy strategies in aquaculture.

## Graphical abstract

Integrated workflow and key findings. 
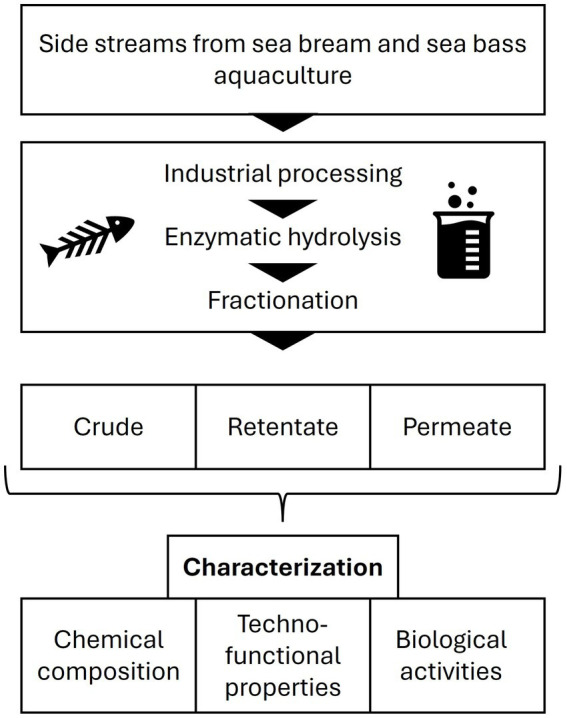

## 1 Introduction

Aquaculture has grown significantly over the past few decades, with species such as sea bream (*Sparus aurata*) and sea bass (*Dicentrarchus labrax*) becoming increasingly popular. However, the processing of these fish generates substantial side streams, including heads, frames, and viscera, which are often underutilized or wasted. Efficient valorization of side streams is essential for sustainable aquaculture and can contribute to the circular economy by converting waste into valuable products (1). Fish side streams contain valuable components, such as proteins, lipids, vitamins, and minerals (2).

Enzymatic hydrolysis is a promising technique for valorizing side streams from aquaculture, as it can break down complex proteins into peptides with various functionalities, such as bioactivities (e.g., antioxidant, anticancer, anti-diabetic) and techno-functional properties (e.g., oil binding, foaming ability, emulsification capacity). The technique is used in today’s food industry to improve different food properties, such as organoleptic properties, digestibility, and bioactivity. Bioactive peptides are usually small, consisting of 2–20 amino acids, with a molecular mass of around 0.4 to 2 kDa (3, 4). They are typically inactive within the natural protein but can be released through processing, such as enzymatic hydrolysis, or through the natural gastrointestinal digestion process. Such peptides can be relevant for a wide range of products, such as nutraceuticals and dietary supplements, as ingredients in functional foods, and for food fortification. Hydrolysis can enhance the biomass’ nutritional value and improve the proteins’ digestibility and bioavailability. Previous studies have demonstrated the potential of enzymatic hydrolysis in producing fish protein hydrolysates with various bioactivities (5, 6). Kaushik et al. reviewed the literature on bioactivity studies of fish protein hydrolysates from 2003 to 2023, showing that most studies evaluated antioxidant activity (45%), followed by ACE inhibitory activity (18%) (7). Few studies exist on the effect of protein hydrolysates on skin cells and wound healing. In addition to potential bioactivities, the techno-functional properties are highly important for the development of food products and ingredients, as they affect food-relevant traits such as texture, appearance, and flavor (7, 8).

Fractionation and filtration are crucial in enhancing fish protein hydrolysates’ functional and bioactive properties. Using membrane filtration with a specific molecular weight cut-off makes it possible to separate the peptides based on their relative molecular sizes, which can influence their bioactivity and potential applications (7, 9). The protein hydrolysate is filtered over a membrane with a specified pore size, producing a permeate (small peptides that pass through the membrane) and a retentate (larger peptides that will not pass through the membrane). The membrane specification can be based on pore size diameter, or molecular weight cut-off (MWCO) (7). Several studies have shown differences in the bioactivity of hydrolysate fractions after fractionation. Often, the low molecular weight peptide fractions exhibit more promising bioactivity than their counterparts of higher molecular weight (10–12). In addition, filtration can up-concentrate the specific bioactive peptides (7), making the fractions more tailored for specific applications in the food and nutraceutical industries. In addition to bioactivity, fractionation also impacts the physicochemical properties of the hydrolysates. By adjusting molecular weight distribution and surface properties such as hydrophobicity, fractionation facilitates the tailoring of protein hydrolysates for specific techno-functional such as emulsifying- and foaming properties which are particularly beneficial in developing functional foods with specific textural and stability characteristics (13–15).

In a previously published article, we compared the chemical, biological, and techno-functional characteristics of protein hydrolysates produced from pre-processed biomass (same as used in the current study) with those produced from unprocessed biomass (16). The dry weight yield was lower and the average molecular weight higher for the hydrolysates produced from pre-processed biomass, indicating that the performed hydrolysis was not as efficient for this biomass and that further studies on hydrolysis conditions and/or other treatments were necessary to bring out the full potential of the biomass. In the current study, we aimed to optimize the hydrolysis process of the pre-processed side streams from farmed sea bream and sea bass and fractionate the hydrolysate to produce two fractions with different peptide sizes. The crude hydrolysate and its fractions, permeate and retentate, were broadly evaluated for chemical characteristics, techno-functional properties, and various bioactivities. This study shows the great potential of hydrolysis and fractionation of protein hydrolysates produced from aquaculture side streams for producing tailored ingredients for foods and nutraceutical products.

## 2 Materials and methods

### 2.1 Biomass, enzymatic hydrolysis and fractionation

Sea bream (*S. aurata*) and sea bass (*D. labrax* L) biomass were provided by Aqua De Mâ (Lavagna, Genova, Italy), consisting of side streams from the processing, mainly skin, heads, bones, and viscera. Some whole fish, which were damaged or too small for sale, were also included. The biomass from Aqua De Mâ was processed at Themis S.p. A. (Milano, Italy) using a patented process (patent number EP 3148683 B1) of grinding, mixing, and drying the biomass, yielding a mixed slurry with low moisture content (17). The proportion of the different fractions (e.g., heads, skin, bones) that were processed is not known. The processed material was shipped to Nofima (Tromsø, Norway) at room temperature and stored at −20°C upon arrival until further use.

The biomass was mixed with water to a final ratio of 1:2 (w/v), using 2 kg biomass and 4 L of water. The hydrolysis was conducted in IKA® LR 1000 Basic reactors (IKA-Werke GmbH & Co. KG, Germany). The mixture was heated to 65°C, followed by the addition of Corolase® 8000 from AB Enzymes GmbH (Darmstadt, Germany) to a final concentration of 0.25% (w/w). The hydrolysis was conducted for 1 h before inactivation for 15 min at 95°C. The mixture was separated through a sieve to remove bones and other large particles before centrifugation at 7000 *g* for 20 min at 20°C (Avanti JXN-26 centrifuge, Beckman Coulter, Brea, California, United States). The oil phase was removed by pipetting, and the hydrolysate was filtered using a Seitz® Depth filter T 2600 (Pall Corporation, New York, United States) and a Whatman No. 4 to remove any remaining oil and particles. The hydrolysis process yielded 3.25 L of hydrolysate, of which 500 mL (15.4%) was frozen and lyophilized, representing the crude sample. The remaining 2,750 mL was fractionated on a PALL CM500SE Centramate 500 S Benchtop Tangential Flow Filtration system (PALL Life Sciences, PALL International Sàrl, Fribourg, Switzerland), equipped with a 3 kDa MWCO filter (T-Series cassettes with Omega™ PES membrane, Cytiva, United States). This resulted in approximately 2.5 L of permeate and 200 mL of retentate. The permeate and retentate were frozen and lyophilized. All samples were lyophilized using a Labconco FreeZone Plus freeze dryer (Labconco Corporation, Kansas City, MO, United States), vacuum-packed, and stored at 4°C until use. The yield was calculated using the following equation:


$$\text{Yield}\;(\%)=\frac{\text{Weight of dried sample}\;(g)}{\text{Weight of}\;\mathrm{wet}\;\text{biomass}\;(g)}*100$$

### 2.2 Chemical characterization

The proximate composition of the crude, permeate, and retentate was characterized. The nitrogen content was analyzed using the Kjeldahl method (NS-EN ISO 5983-2), and crude protein content was estimated based on N x 6.25. The dry matter content was determined by drying at 103°C (ISO 6496) and ash by combustion at 550°C (ISO 5984). The lipid content was determined by acid hydrolysis using the EC No 152/2009 method. Total amino acid content was determined by High Performance Liquid Chromatography (HPLC) with fluorescence detection, as described previously (18), with minor modifications. The run time was 32 min with a 0.4 mL/min flow. Peptide size distribution and average molecular weight (AMW) was determined by Size Exclusion Chromatography (SEC) as described in detail previously (19), using peptide standards ranging from 0.2 to 29 kDa.

A color analysis was performed on the crude, permeate, and retentate samples. A double-beam UV–visible spectrophotometer (Agilent Cary 100 Varian Co., Santa Clara, CA, United States) equipped with an integrating sphere (Varian DRA) that could uniformly disperse light throughout all interior surfaces at a resolution of 1 nm was operated to measure the color in the range of 300–900 nm. By employing a white Spectralon® disk as a reference, triplicate analyses were performed. The CIE D65 illuminant was used to automatically derive the CIELab coordinates L* (lightness), a* (greenish-reddish), and b* (bluish-yellowish) from the raw spectral data using the Cary100 Color program. The samples were compared to each other, and Delta E was calculated as follows:


$$\mathrm{\Delta E}=\sqrt{{\left(L2-L1\right)}^{2}+{\left(a2-a1\right)}^{2}+{\left(b2-b1\right)}^{2}}$$

By Attenuated Total Reflectance Fourier-Transform Infrared (ATR-FTIR) spectroscopy, a qualitative determination was conducted using an FT-IR spectrophotometer (Perkin Elmer, Inc., Waltham, MA, United States). Background was recorded before measurements, then spectra were acquired at room temperature from 4,000 to 600 cm^−1^ at a resolution of 4 cm^−1^, accumulating 4 scans per sample.

### 2.3 Techno-functional characterization

#### 2.3.1 Foaming properties

Before characterization of techno-functional properties, the crude, permeate, and retentate samples were sieved through a 400 μm mesh size sieve (6016000400, Serial No. 7264339, Retsch UK Ltd., Derbyshire, United Kingdom) to standardize the particle size. To determine the foaming properties, ~0.2 g freeze-dried sample (*n* = 3) was mixed with 0.1 M sodium phosphate buffer (13.5 mL). The solution was carefully transferred to a 50 mL measuring cylinder, and the initial volume was recorded. Foam was generated at 18,000 rpm for 30 s using an Ultra Turrax (T25, IKA, Staufen, Germany) fitted with a S25N-10G dispenser. The total volume, including the foam, was recorded immediately after homogenization. The foam height was monitored at predetermined intervals (0, 0.5, 1, 1.5, 2, 5, 10, 15, and 20 min). The foam volume was calculated as the difference between the total and liquid-phase volumes. Foaming capacity (%) and foam stability (%) were calculated as follows:


$$\begin{array}{l} \text{Foaming capacity}\;(\%)=\\ \frac{\text{Total volume after homogenisation}\;(\mathrm{mL})-\text{Initial volume}\;(\mathrm{mL})}{\text{Initial volume}\;(\mathrm{mL})}*100 \end{array}$$

$$\begin{array}{l} \text{Foaming stability}\;\mathrm{at}\\ \;t=i\;\min\;(\%)=\frac{\text{Foam volume}\;(\mathrm{mL})\;\mathrm{at}\;t=i\;\min\;}{\text{Foam volume}\;(\mathrm{mL})\mathrm{at}\;t=0\;\min\;}*100 \end{array}$$

#### 2.3.2 Emulsifying properties

The emulsifying properties of the crude, permeate, and retentate samples were evaluated by dissolving 0.2 g of freeze-dried sample (*n* = 3) in 10 mL of 0.1 M sodium phosphate buffer. The solution (3 mL) was mixed with 1 mL of locally purchased canola oil (Eldorado, Until AS, Oslo, Norway) using a Gilson™ Microman™ M1000 pipette (Gilson, VWR International, Norway) and homogenized at 20,500 rpm for 1 min using Ultra Turrax (T25, IKA, Staufen, Germany) fitted with a S25N-10G dispenser. Immediately after homogenization, the sample solution was mixed with 0.3% SDS buffer at a 1:100 ratio (v/v). The absorbance of the samples was measured at 0 and 30 min at 500 nm by using a microplate reader (Synergy H1, Biotek Instruments, Winooski, VT, United States). Emulsifying activity index (EAI) and emulsifying stability index (ESI) were determined in triplicate as follows:


$$\mathrm{EAI}(m^{2}/g)=\frac{\left(2.303\times 2\times A_{o}\times \text{Dilution}\right)}{\left(\varphi \times C\times 10000\right)}$$

$$\mathrm{ESI}\;(\min)=(\frac{A_{o}}{A_{o}-A_{t}})\times (t_{\min})$$

where A₀ and A_t_ are the absorbance values at t = 0 min and 30 min, respectively. ‘*φ*’ is the oil volume fraction (0.25) and ‘C’ is the concentration of the lyophilized hydrolysate (g/mL) aqueous phase. Dilution is 100 based on the 1:100 dilution with SDS. t_min_ is the predefined incubation time (30 min).

#### 2.3.3 Oil binding capacity

The crude, permeate, and retentate samples were evaluated for oil binding capacity. 0.2 grams freeze-dried samples (n = 3) were mixed with 2 grams of canola oil (Eldorado, Until AS, Oslo, Norway) using Gilson™ Microman™ M1000 pipette. The samples were vortexed at maximum speed for 30 s followed by 5 min settling, with an additional 15 s of vortexing before centrifugation. Samples were centrifuged at 2500 g for 30 min at 20°C (Hettich ROTINA 420R). Using a Gilson pipette, the supernatant was carefully transferred to a new tube and weighed to calculate the oil binding capacity expressed as gram oil retained per gram of hydrolysate:


$$\begin{array}{l} \text{Oil binding capacity}=\\ \frac{\text{Weight of oil added}\;(g)-\text{Weight of supernatant}\;(g)}{\text{Weight of hydrolysate sample}\;(g)} \end{array}$$

### 2.4 Biological activity characterization

#### 2.4.1 ACE inhibitory activity

The crude, permeate, and retentate samples were evaluated for ACE inhibitory activity. The ACE-inhibitory assay was measured spectrophotometrically using Furylacryloyl-Phenylalanyl-Glycyl-Glycine (FAPGG) as substrate, as described previously (20), with some modifications. Briefly, 10 μL of ACE solution (0.25 unit/mL distilled water) and 10 μL of each sample solution were placed in one well of a 96-well microtiter plate. The microplate was immediately transferred to the microplate SpectraMax M5 spectrophotometer (Molecular Devices LLC, United States) tempered at 37°C. A sample of 150 μL substrate solution (0.88 mM FAPGG in 50 mM Tris–HCl, pH 7.5, containing 0.3 M NaCl), preheated to 37°C, was placed into each well to start the reaction. The absorbance at 340 nm was recorded every 60 s for 60 min. The control was prepared using 10 μL of buffer (50 mM Tris– HCl, pH 7.5, containing 0.3 M NaCl) instead of hydrolysate. Triplicate samples and controls were used in these studies. Negative control without sample, and positive control with a recognized ACE-inhibitor (captopril, C4042 Sigma Aldrich) were used. The ACE activity was determined from the average slope of the absorbance vs. time curve in its linear section, and the ACE inhibition (%) was calculated using the equation below:


$$\mathrm{ACE}\;I\;\text{inhibition}\;(\%)=[1-(\frac{pA_{\text{inhibitor}}}{pA_{\text{control}+}})]\cdot 100$$

where pA_inhibitor_ and pA_control_ are the slopes determined by the hydrolysate and control samples, respectively. The concentration-response curves were obtained by assaying various dilutions of the samples and plotting the ACE inhibition percentage as a function of concentration. The concentration causing 50% ACE inhibition (IC_50_), obtained from triplicate samples, was calculated using the mathematical models proposed by Estévez (21). Results were expressed as means ± SD. *p* values were determined by a paired Student’s t-test. The difference was considered significant when *p* < 0.05.

#### 2.4.2 Cellular antioxidant activity

The cellular antioxidant assay (CAA) was performed using the human liver hepatocellular carcinoma cell line Hep G2 (ATCC HB-8065). Hep G2 cells were grown in Eagle’s Minimum Essential Medium (EMEM) (ATCC-30-2003) supplemented with 10% FBS (fetal bovine serum), antibiotic (solution of 10,000 units of penicillin and 10 mg streptomycin/mL) at 1%. The cultures were maintained in a 37°C incubator with 5% CO_2_ and a humid atmosphere. Firstly, cell viability was evaluated in the presence of the samples to be tested to determine which concentrations to choose for the CAA. Hep G2 cells were seeded at a concentration of 5×10^3^ cells/well of growth medium on a 96-well plate, and incubated for 24 h at 37°C. The cells were washed with phosphate-buffered saline (PBS), and different concentrations of the samples to be tested were applied to the cells. An equal volume of medium was used as the control treatment. The plates were incubated at 37°C for 24 h before a methyl thiazoyl terazolium (MTT) assay was conducted. The cell viability was calculated as percent viability with respect to the control. The MTT assay is based on NAD(P)H-dependent cellular oxidoreductase enzymes that can reduce the tetrazolium dye, MTT (3-(4,5-dimethylthiazol-2-yl)-2,5-diphenyltetrazolium bromide), to insoluble formazan. A dimethyl sulfoxide (DMSO) solution is added to dissolve the formazan product into the solution, and the absorbance is measured spectrophotometrically at 570 nm. Serial dilutions of samples were used for the CAA, starting with the highest dilution that did not reduce cell viability.

After the viability assay, the intracellular antioxidant capacity of the crude, permeate, and retentate was assessed using the CAA as described previously (22), with some modifications. Briefly, Hep G2 cells were grown in EMEM with 10% FBS, 100 μg/mL streptomycin, and 100 units/mL penicillin. During the logarithmic growth phase, cells at a concentration of 6 × 10^5^ cells/mL were seeded into a 96-well microplate (100 μL/well) and incubated for 24 h in a fully humidified atmosphere with 5% CO_2_ at 37°C. The cells were washed with PBS and incubated for an additional 2.5 h in a medium containing diosmetin and 2′,7′-dichlorofluorescein diacetate (DCFH-DA) at a final concentration of 25 μM, along with different concentrations of each test sample as determined by the viability assay. Next, the cells were washed with PBS, and 100 μL of a medium containing Hank’s balanced salt solution (HBSS) with 10 mM HEPES and 500 μM 2,2′-Azobis(2-amidinopropane) dihydrochloride (AAPH, Sigma Aldrich, from Merck KGaA, Darmstadt Germany) was added to each well. The 96-well microplate was placed in a SpectraMax M5 spectrophotometer (Molecular Devices LLC, United States) at 37°C, and the fluorescence (λ_ex/em_, 485/520 nm) was monitored every 5 min for 1 h. Each plate included triplicate controls and blank wells. Control wells contained cells treated with DCFH-DA and oxidant AAPH, while blank wells contained cells treated with dye and HBSS without oxidant. In addition, a positive control was included, which, instead of the sample, contained a compound with recognized cellular antioxidant capacity, quercetin (Q4951, Sigma Aldrich, from Merck KGaA, Darmstadt Germany). The fluorescence value was calculated using the method of Liao. All results were presented as % inhibition of oxidation (mean ± standard error).

#### 2.4.3 Anti-inflammatory capacity

The anti-inflammatory assay was performed using the murine monocytes/macrophage cell line RAW 264.7 (ATCC TIB-71). Cells were cultured in Dulbecco’s Modified Eagle Medium (DMEM; ATCC-30-2002) supplemented with 10% heat-inactivated FBS and antibiotic (solution of 10,000 units of penicillin and 10 mg streptomycin/mL) at 1%. They were maintained at 37°C with a 5% CO_2_ atmosphere. Firstly, cell viability was evaluated in the presence of the crude, permeate, and retentate samples. RAW cells were seeded at 5×10^4^ cells/well in a growth medium on a 96-well plate and incubated for 24 h at 37°C. The cells were washed with PBS, and different concentrations of the samples were applied to the cells. An equal volume of medium was used as the control treatment. The plates were incubated at 37°C for 24 h, and the MTT assay was conducted as described in section 2.4.2. The cell viability was calculated as percent viability with respect to the control. For each sample, the highest concentration that did not affect cell viability was selected further for the anti-inflammatory assay.

The selected concentrations of hydrolysates were tested to verify their influence in the *in vitro* anti-inflammatory assay. Exponentially growing RAW 264.7 macrophages were plated in 96-well plates at a density of 2×10^5^ cells/ well in 100 μL of culture medium, allowing them to adhere overnight. Cells were then stimulated with 100 ng/mL lipopolysaccharide (LPS), and the plates were incubated for 30 min under optimal culture conditions. Subsequently, cells were treated with 100 μM hydrocortisone (positive control) or samples dissolved in culture medium at the selected concentration for each sample and incubated at 37°C with 5% CO_2_ for 24 h. In addition, cells were maintained as LPS control without treatment and media control without LPS or treatment. Cell-free supernatants were collected and used to measure nitrogen oxide (NO) and Interleukin-6 (IL-6) contents. NO content was determined using the Griess reaction kit (Griess reagent kit, Invitrogen Thermo Fisher, Massachusetts, United States), while Enzyme-linked immunosorbent assay (ELISA) kits were used for IL-6 cytokine determination (IL-6 Mouse ELISA Kit; Invitrogen Thermo Fisher, Massachusetts, United States). The kits were used following the manufacturer’s recommendations.

For nitrites and IL-6, the percentage of inhibition in the production of inflammatory factors by macrophages was calculated for each treatment group using the following equation:


$$\text{Inhibition}\;(\%)=\frac{1-[\text{factor}]\text{sample}}{[\text{marker}]\text{control}\;\mathrm{LPS}}*100$$

where [factor] sample is the concentration of the inhibition factor studied (NO or IL-6) obtained in the sample, while [marker] is the concentration of the inhibition factor studied obtained in the LPS control. Results were expressed as means ± SD. *p* values were determined by paired Student’s t-test. The difference was considered significant when *p* < 0.05.

#### 2.4.4 Anti-osteoporotic capacity

The anti-osteoporotic assay explores the influence of compounds or complex samples on bone growth *in vitro*. In the present study, the ability of the crude, permeate, and retentate to promote the proliferation and differentiation of osteoblast cells of bone-like matrix on mouse MC3T3-E1 osteoblastic cell subclone 4 (ATCC CRL-2593) was evaluated. Also, the capacity to inhibit the osteoclast proliferation on murine monocyte–macrophage cell line RAW 264.7 (ATCC TIB-71) cells was evaluated *in vitro*. This is expected to give leads about the potential usefulness of hydrolysates as effective anti-osteoporotic agents. The assay includes a proliferative assay of the MC3T3 cell line, a proliferative assay of the RAW 264.7 cell line, and a differentiation assay of the MC3T3 cell line. Cell proliferation was measured by the MTT assay as described in section 2.4.2. Before treatment with the test samples, cells were seeded in 96-well plates at 5×10^3^ cells/well for MC3T3-E1 cell line and 2×10^4^ cells/well for RAW 264.7 cell line. The plates were cultured for 24 h in optimal conditions of growth: minimum alpha essential medium (αMEM, Gibco, Thermo Fisher Scientific, United States) for MC3T3-E1 cell line and DMEM media for RAW 264.7 cell line, supplemented with 10% heat-inactivated FBS, and 1% of antibiotic solution (10,000 units of penicillin and 10 mg streptomycin/mL) in 5% CO_2_ at 37°C. The media were replaced with a fresh medium containing the test samples to evaluate proliferative efficiency. After treatment with the test samples for 24 h, the MTT assay was performed. The proliferation of cells in the medium without the test compound was defined as 100% for a comparative standard.

For the bone cell differentiation assay, MC3T3-E1 osteoblastic cell subclone 4 (ATCC CRL-2593) was cultured in basal media (BM). The BM was αMEM supplemented with 10% heat-inactivated FBS, and 1% antibiotic solution (10,000 units of penicillin and 10 mg streptomycin/mL). The cells were maintained at 37°C in a 5% CO_2_ atmosphere. The MC3T3-E1 cells (5×10^4^ cells/mL/well) were pre-cultured in BM for 24 h in 24-well plates. The medium was then changed to osteogenic media (OM) with or without the test samples on day 0 and was renewed every 2 days until the end of the experiment. The OM media consisted of BM supplemented with 50 μg/mL ascorbic acid, 5 mM *β*-glycerophosphate, and 10 nM dexamethasone (23, 24). Changes in alkaline phosphatase (ALP) activity were quantified because these changes reflect mature osteoblast formation and have been accepted as an early osteoblast activity marker (25). ALP activity was determined in cell lysates obtained by treatment with lysis buffer (0.5% Triton X-100 in 50 mM Tris–HCl buffer, 150 mM NaCl, pH 7.4, 400 μL/well) for 30 min at room temperature. The cell lysate was collected separately, centrifuged at 13,000 g for 15 min, and supernatants were stored at −20°C. The ALP activity was determined by the Alkaline Phosphatase Activity Colorimetric Assay Kit (K412-500, BioVision, Milpitas, United States) following the manufacturer’s recommendations. The activity was calculated as nmol of p-NP produced/mg protein/min and expressed as % relative ALP activity compared with the control. To normalize the differences in protein content between wells, the protein concentration of each well was determined by Pierce protein assay (Thermo Fisher Scientific, Massachusetts, United States) (24). Results were expressed as means ± SD. *p* values were determined by paired Student’s t-test. The difference was considered significant when *p* < 0.05.

#### 2.4.5 Hepatoprotective effect

The hepatoprotective assay was performed using the liver cell line Hep G2 (ATCC® HB-8065™). Cells were cultured in EMEM (ATCC-30-2003) supplemented with 10% of heat-inactivated FBS, an antibiotic (solution of 10,000 units of penicillin and 10 mg streptomycin/mL) at 1%, and were maintained at 37°C and 5% CO_2_. Firstly, the cell viability in the presence of the samples was assessed. Hep G2 cells were seeded at a concentration of 5×10^3^ cells/well of growth medium in a 96-well plate, and incubated for 24 h at 37°C. The cells were washed with PBS and treated with different concentrations of test samples. An equal volume of medium was used as the control treatment. The plates were incubated at 37°C for 24 h, then the MTT assay was conducted (see section 2.4.2), and the cell viability was calculated as % of viability with respect to the control. For each hydrolysate, the highest concentration that did not affect cell viability was selected for the hepatoprotective screen.

The selected concentrations of hydrolysates were tested to verify their influence on the *in vitro* hepatoprotective assay. Exponentially growing Hep G2 cells were plated in 96-well plates at a density of 5×10^3^ cells/well in 100 μL of culture medium, allowing them to adhere overnight. Cells were then stimulated with samples dissolved in culture medium and incubated at 37°C, 5% CO_2_ for 24 h. Control wells were maintained without treatment, only with culture media. Then, cells were washed twice with PBS (without Ca^2+^ and Mg^2+^). They were subsequently treated with culture medium EMEM without FBS, supplemented with a mixture of oleic and palmitic fatty acids (proportion 2:1,1 mM) and 1% bovine serum albumin (BSA). A control, without sample, and a control of each sample, without fatty acids, were maintained to evaluate the effect of the sample itself on the accumulation of fatty acids in the cells, these were incubated with the same volume of culture medium EMEM without FBS and 1% BSA. The plates were incubated again at 37°C for 24 h and after which the accumulation of lipids in the cells was analyzed with AdipoRed™ Assay Reagent (Lonza, Switzerland), following the manufacturer’s instructions. Briefly, the wells were washed twice with 200 μL PBS, and then 5 μL Adipored reagent was added to 200 μL PBS. After 10 min, fluorescence was measured with excitation at 485 nm and emission at 572 nm with a microplate reader (Molecular Devices LLC, United States). The results were calculated as an average of the relative percentage of fatty acid accumulation with respect to the control without the sample. Results were expressed as means ± SD. *p* values were determined by paired Student’s t-test. The difference was considered significant when *p* < 0.05.

#### 2.4.6 Cell viability and wound healing activity

The effect of the crude, permeate, and retentate samples on the cell viability of the human keratinocyte cell line (HaCaT, Cytion Eppelheim, Germany) was assessed using the MTT assay. The HaCaT cells were cultured in controlled conditions (37°C and 5% CO_2_) in flask T-75, using DMEM (Euroclone, Pero, Italy) supplemented with 1% L-Glutamine and 10% FBS, until 80% confluence was obtained. The cells were washed with 4 mL PBS and detached using 2 mL Trypsin. 75,000 cells/mL were seeded in a 96-well plate and incubated for 24 h. After incubation at 37°C with 5% CO_2_, the cells were exposed to different concentrations of samples, followed by a 24-h incubation. Different concentrations of samples were prepared: 0.05, 0.025, and 0.015 mg/mL, diluting the stock solution in DMEM without supplements. After incubation with samples for 1 day, the cell viability was assessed using the MTT assay. MTT reagent was solubilized in PBS at a concentration of 5 mg/mL and diluted in culture medium to a final concentration of 0.5 mg/mL. Subsequently, 200 μL of MTT solution was added to each well and incubated for 3 h. Before reading the plate, the obtained formazan crystals were solubilized in acid-alcohol (0.04 N HCl in 2-propanol) solution. Absorbance was measured at 570 nm using a microplate reader (Byonoy Absorbance 96, Germany).

The wound healing capabilities of the crude, permeate, and retentate were assessed on human keratinocytes using the T-scratch assay. The assay mimics cell migration during wound closure in the presence of different assayed compounds or samples (26). For the T-scratch assay, three different concentrations were tested based on the results from the initial cell viability assay: 0.05, 0.025, and 0.015 mg/mL, diluted in DMEM. Negative control was prepared using cells treated with only DMEM without serum. The cells were seeded in a 12-well plate containing the Ibidi tile positioned in the center of the well (Ibidi GmbH, Gräfeling, Germany), at a density of 572,000 cells/mL, and incubated (37°C with 5% CO_2_) for 24 h in complete DMEM. Following incubation, the inserts and the medium were removed, and the wells were washed with PBS. Cell migration was monitored, and images were captured at 0 h and 24 h using the MATEO microscope with 4 × magnification (Leica microsystems, Wetzlar, Germany) and analyzed using ImageJ-win32 Wound_healing_size_tool_updated macro (27). The results are reported as the percentage of distance compared to the area before and after the treatment. The wound closure was calculated using the following equation:


$$\text{Wound closure}\;(\%)=\frac{\mathrm{AT}0-\mathrm{AT}24}{C.\mathrm{AT}0-C.\mathrm{AT}24}\;x\;100$$

where A_T0_ is the scratch area of the sample-treated cells at time 0, A_T24_ is the scratch area of the sample-treated cells after 24 h, C. AT_0_ is the scratch area of the control cells at time 0, and C. AT_24_ is the scratch area of the control cells after 24 h.

## 3 Results and discussion

### 3.1 Yield and chemical characterization

#### 3.1.1 Yield

In total, 2 kg of biomass was hydrolyzed in a 1:2 ratio (w/v) with water using Corolase® 8000. This resulted in 3.25 L of hydrolysate and about 450 mL of oil after separation by centrifugation. The calculated total wet weight yield was 20.4%. In a previous study, we reported a wet weight yield of 6.53%, using the same biomass and enzyme, but with a 1:1 biomass-to-water ratio (16). This indicates that the amount of water used during hydrolysis significantly increases the hydrolysis yield, which is understandable as the biomass is dehydrated and therefore contains much less water compared to more commonly used residual materials. A fraction of the hydrolysate, 500 mL (15.4% of total volume), was freeze-dried directly, yielding 62.83 g of crude sample. The remaining 2,750 mL of crude hydrolysate (84.6% of total volume) was fractionated using a 3 kDa MWCO filter, resulting in 2.5 L permeate and 200 mL retentate, yielding 239.37 g and 73.53 g, respectively. The total wet weight yield for the permeate and retentate was 14.1 and 4.3%, respectively. The crude, permeate, and retentate were characterized with respect to chemical, techno-functional, and biological activity properties, as described in the following sections.

#### 3.1.2 Proximate composition

The proximate composition of the pre-processed sea bream and sea bass side streams have been published previously (16), displaying low moisture content and high oil and protein content. In detail, the moisture content was 1.8%, oil content 53.2%, protein 41.9%, and ash content 7.4%. The proximate composition of the crude, permeate, and retentate samples are shown in Table 1. The analyses showed that all samples had high protein contents, above 83% (measured by Kjeldahl, N x 6.25). As expected, the ash content was higher in the crude and permeate compared to the retentate, indicating that the salts passed through the membrane into the permeate during fractionation. Meanwhile, the oil content was higher in the retentate than the crude and permeate, indicating that the membrane retains it.

**Table 1:** Proximate composition of crude, permeate, and retentate protein samples (protein *n* = 2, ash *n* = 1, oil *n* = 1, moisture *n* = 1).

Composition	Crude	Permeate	Retentate
Protein (%)^a^	84.7	83.6	86.2
Ash (%)	8.1	8.2	4.1
Oil (%)	<1.5^b^	<1.5^b^	4
Moisture (%)	7	7	3.5

a Allowed replicate variation: Protein: ≤0.8%.

b Oil content reported down to 1.5% due to measurement uncertainty.

#### 3.1.3 Amino acid composition

The total amino acid compositions of the crude, permeate, and retentate samples were analyzed (Table 2). The total sum of amino acids was higher in the retentate (82.4%) compared to the crude (77.6%) and permeate (75.9%), as was also observed in the Kjeldahl protein analyses. The sum of essential amino acids was slightly higher for the crude and the permeate, while the sum of dispensable amino acids was higher for the retentate. The collagen-associated amino acids, hydroxyproline, proline, and glycine, were found in higher levels in the retentate compared to the permeate and crude samples. The most abundant amino acids found in the samples were glutamic acid and glycine, which correspond to previous analyses of sea bass and sea bream side streams and hydrolysates (12, 16, 28–30).

**Table 2:** Total amino acid composition (g/100 g) of the crude, permeate, and retentate samples (*n* = 2) and results reported as mean values with specified allowed replicate variation^a^.

Amino acids	Crude	Permeate	Retentate
EEA^b^			
Arg	5.5	5.9	4.8
His	1.3	1.3	1.4
Ile	2.7	2.6	2.7
Leu	5.1	5.3	4.4
Lys	5.9	6.1	5.3
Met	2.2	2.2	2.0
Phe	2.6	2.7	2.4
Thr	3.4	3.3	3.7
Val	3.5	3.5	3.4
Sum EEA	32.2	32.9	30.1
DAA^c^			
Ala	6.2	6.3	6.0
Asp	7.1	6.6	8.4
Glu	11.3	10.9	12.2
Gly	8.5	7.9	10.4
H-pro	2.1	1.8	3.0
Pro	5.2	4.6	6.9
Ser	3.6	3.5	4.1
Tyr	1.4	1.4	1.3
Sum DAA	45.4	43.0	52.3
Sum AA	77.6	75.9	82.4
EEA/AA (%)	41.5	43.3	36.5

a Allowed replicate variation: Relative standard deviation ≤6% for 2/3 of amino acids.

b Essential amino acids.

c Dispensable amino acids.

#### 3.1.4 Size exclusion chromatography

The AMW and peptide size distribution of the crude, permeate, and retentate were assessed using SEC, and a difference between the samples was observed (Table 3). As expected, the AMW was highest for the retentate sample (1771.5 Da), followed by the crude (1063.0 Da), and lastly the permeate (710.0 Da). The same trends can be observed from the peptide size distribution, showing a higher relative abundance of larger peptides (>2000 Da) in the retentate sample. In our previous study, we obtained an AMW of 3364 Da when hydrolyzing the pre-processed biomass using the same enzyme but a 1:1 biomass-to-water ratio (16). As observed for the hydrolysate yield, the biomass-to-water ratio also greatly impacts the size of the peptides in the hydrolysate.

**Table 3:** Average molecular weight (AMW, Da) and peptide size distribution of the crude and fractionated samples, measured by HPLC-SEC.

AMW/peptide size distribution		Crude	Permeate	Retentate
AMW (Da)		1063.0 ± 4.2	710.0 ± 1.4	1771.5 ± 9.2
Peptide size distribution (Da)	0–500	28.5 ± 0.0	35.6 ± 0.2	16.2 ± 0.0
	500–1000	37.0 ± 0.0	38.4 ± 0.1	31.7 ± 0.1
	1000–2000	25.7 ± 0.1	22.9 ± 0.1	31.1 ± 0.1
	2000+	8.9 ± 0.1	3.2 ± 0.0	21.0 ± 0.0

Peptide size distributions are divided into size-based fractions, as a percentage of the total. Results are reported as mean values with standard deviation (*n* = 2).

#### 3.1.5 Color analysis

Color is an important parameter to be examined, as it is probably the first factor considered by the consumers (31). It is a crucial factor in determining the acceptability of protein hydrolysates, particularly when they are intended for inclusion in functional foods. Conversely, the color of protein hydrolysates is generally less significant when used as ingredients in dietary supplements, as they are often encapsulated or blended with other components that can modify and/or mask their color (32).

**Figure 1:**
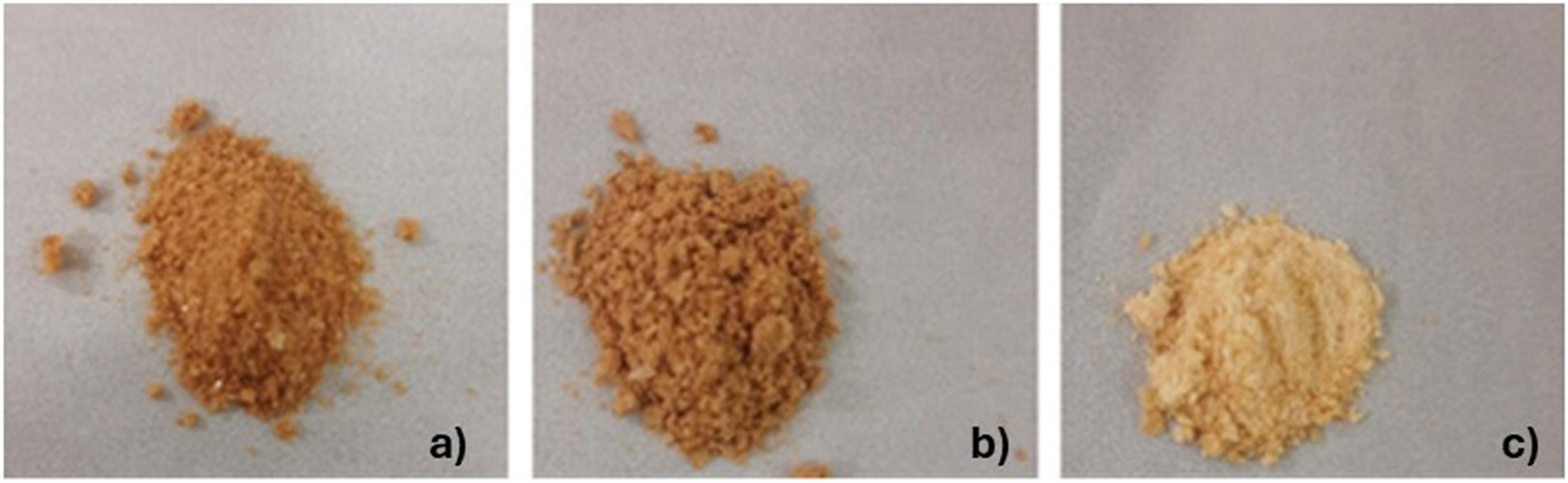
Crude **(a)**, retentate **(b)**, and permeate **(c)** samples.

The crude, permeate, and retentate samples (Figure 1) were investigated for their CIE Lab color profiles (Table 4), indicating the parameters lightness (L*), redness (a*), and yellowness (b*). The brown-reddish color of the crude and retentate samples can be attributed to the influence of astaxanthin, which is an antioxidant pigment present in the flesh of fish together with a residue of lipids (33), which is concentrated in the retentate sample, but also to the presence of oxidized myoglobin (34). As can be visibly observed, the lightness (L*) is very similar between crude (50.66) and retentate (50.34) but increases significantly in the permeate (65.81), indicating that enzymatic hydrolysis facilitated the unfolding of protein structures, leading to smaller particles with larger surface areas and improved light dispersion (32). The redness (a*), on the other hand, decreases from 10.90 for crude, then 8.90 for retentate, to 4.69 for the permeate. As for the yellowness (b*), similar results are reported for retentate (20.06) and permeate (19.20), while a higher result was obtained for the crude (23.52). From the obtained values of L*, a* and b*, a color table was created by converting the CIEL*a*b* color space to the red/green/blue (RGB) scale, by CIELAB color space & coordinates conversion (Table 4) (35).

**Table 4:** CIELab parameters of crude, retentate, and permeate samples (*n* = 5).

CIE	Crude	Retentate	Permeate
L*	50.66 ± 0.02	50.34 ± 2.13	65.81 ± 0.37
a*	10.90 ± 0.08	8.90 ± 0.74	4.69 ± 0.03
b*	23.52 ± 0.17	20.06 ± 1.75	19.20 ± 0.37
RGB	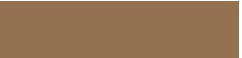	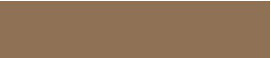	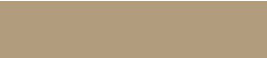

The results are presented as mean values with standard deviation. L* represents lightness, a* represents redness, and b* represents yellowness.

The obtained values are comparable to those obtained by Klomklao et al. on protein hydrolysates from tuna viscera, which appeared yellow-brown in color (L* = 62.46, a* = 1.44, and b* = 23.05), and to those obtained by Sathivel et al. on yellowish enzymatic hydrolysates from salmon (L* = 40.1–43.3, a* = 8.1–10.2, and b* = 22.3–30.8) (36, 37). Furthermore, in a previous article, the authors obtained protein hydrolysates from a mixed biomass of cooked tuna, which exhibited a lighter and more yellow color (38), highlighting how the color of the protein hydrolysates is closely related to the starting biomass besides extraction/purification steps. A comparison was made between the three samples in terms of Delta E (Table 5), showing a high similarity for crude and retentate with a low value of Delta E (3.29), and a mismatch between permeate and the others (16.63 and 13.64 for crude and retentate, respectively). The results match what can be visually observed by looking at the samples (Figure 1).

**Table 5:** Delta E for crude, retentate and permeate.

Delta E	Crude vs. retentate	Crude vs. permeate	Retentate vs. permeate
ΔE	3.29	16.63	13.64

#### 3.1.6 ATR-FTIR analysis

**Figure 2:**
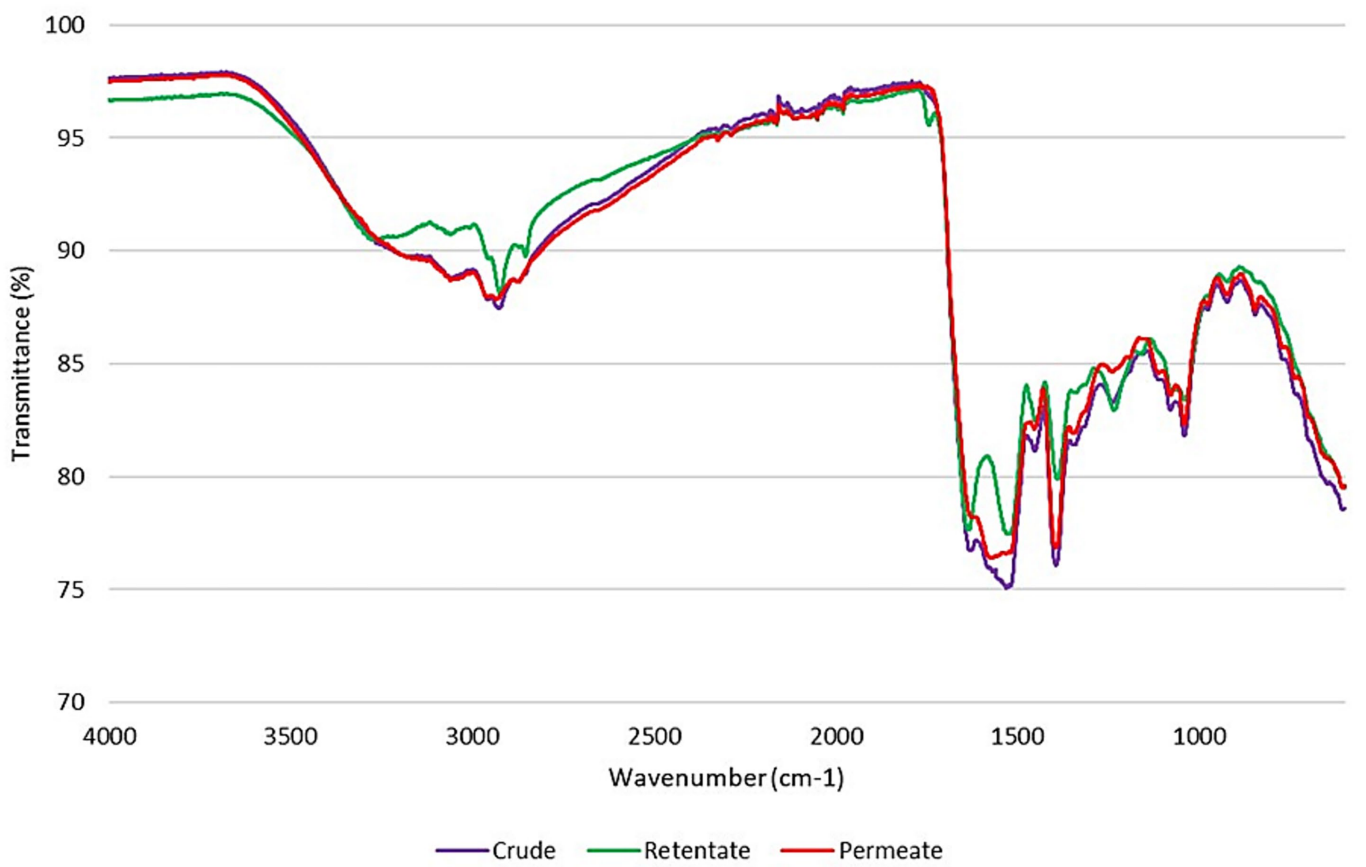
FTIR spectra of crude, permeate, and retentate samples.

ATR-FTIR spectroscopy is a non-destructive analytical technique that can be applied to study the structure of proteins or peptides (39). The spectra of the protein hydrolysates are reported in Figure 2. The most relevant peaks, defining protein structure, derive from the absorptions of amide groups that bind amino acids. The Amide A (3500–3000 cm^−1^), which is linked to the OH- and NH-stretching (40), is present as a band in all the protein hydrolysates, with a most prominent peak for the retentate at 3266 cm^−1^. The absorptions associated with the Amide B peaks (3000–2900 cm^−1^), generated by asymmetric stretching of NH_3_ and = CH (41), are located at 2926/2923/2935 cm^−1^ for crude, retentate, and permeate. These peaks provide information about the protein’s secondary structure and the possible presence of lipid contamination in the sample. A more prominent peak for the retentate could be related to the higher amount of oil in the protein sample (Table 1). Moreover, a more evident peak at 1740 cm^−1^ in retentate than in the others, could be also related to the C=O stretching of lipids. The Amide I peaks (between 1700 and 1600 cm^−1^), generated from the stretching of C=O with weak C-N stretch and N-H bending vibrations, are found at 1628/1633/1625 for crude, retentate, and permeate samples, respectively, with less intense peaks for the crude and the permeate samples most likely due to the presence of smaller or denatured peptides (lower AMW), unlike the retentate sample in which the highest molecular weight peptides are concentrated. Glassford et al. noted that shifts in the Amide I band are linked with the protein’s secondary structure (42). Specifically, *β*-sheet structures typically exhibit bands between 1620 and 1640 cm^−1^, suggesting this as the dominant structure for these protein samples even in the permeate, where the Amide I peak overlaps with the lower frequency Amide II-band. In addition, the enzyme and the reaction temperature influence the secondary structure content of proteins (43) and can also affect the location of Amide II. The peak of amide II, attributed to in-plane bending of NH and CN stretching, usually appears at 1600–1500 cm^−1^, and for these protein samples, the peaks are found at 1532/1529/1550 cm^−1^ for crude, retentate and permeate, respectively. As for the amide I peaks, also the amide II peaks follow the same intensity trend, that is, less intense for permeate and crude, compared to the retentate peak due to their molecular weight distributions. The Amide III band is caused by a combination of CN stretching, NH bending, and deformation vibrations of CH and NH (42), found at 1239/1235/1241 cm^−1^ for crude, retentate and permeate, respectively.

From these outcomes, it is possible to state that the retentate has a more organized and stable structure, with more prominent peaks corresponding to the amide bands (Amide A, B, I, II, III), indicating proteins with higher molecular weight and a more defined secondary structure. On the contrary, in the crude and permeate samples, the less intense peaks in the amide bands indicate the presence of denatured proteins or proteins with lower molecular weight, as highlighted by HPLC-SEC analysis (Table 3).

### 3.2 Techno-functional characterization

**Figure 3:**
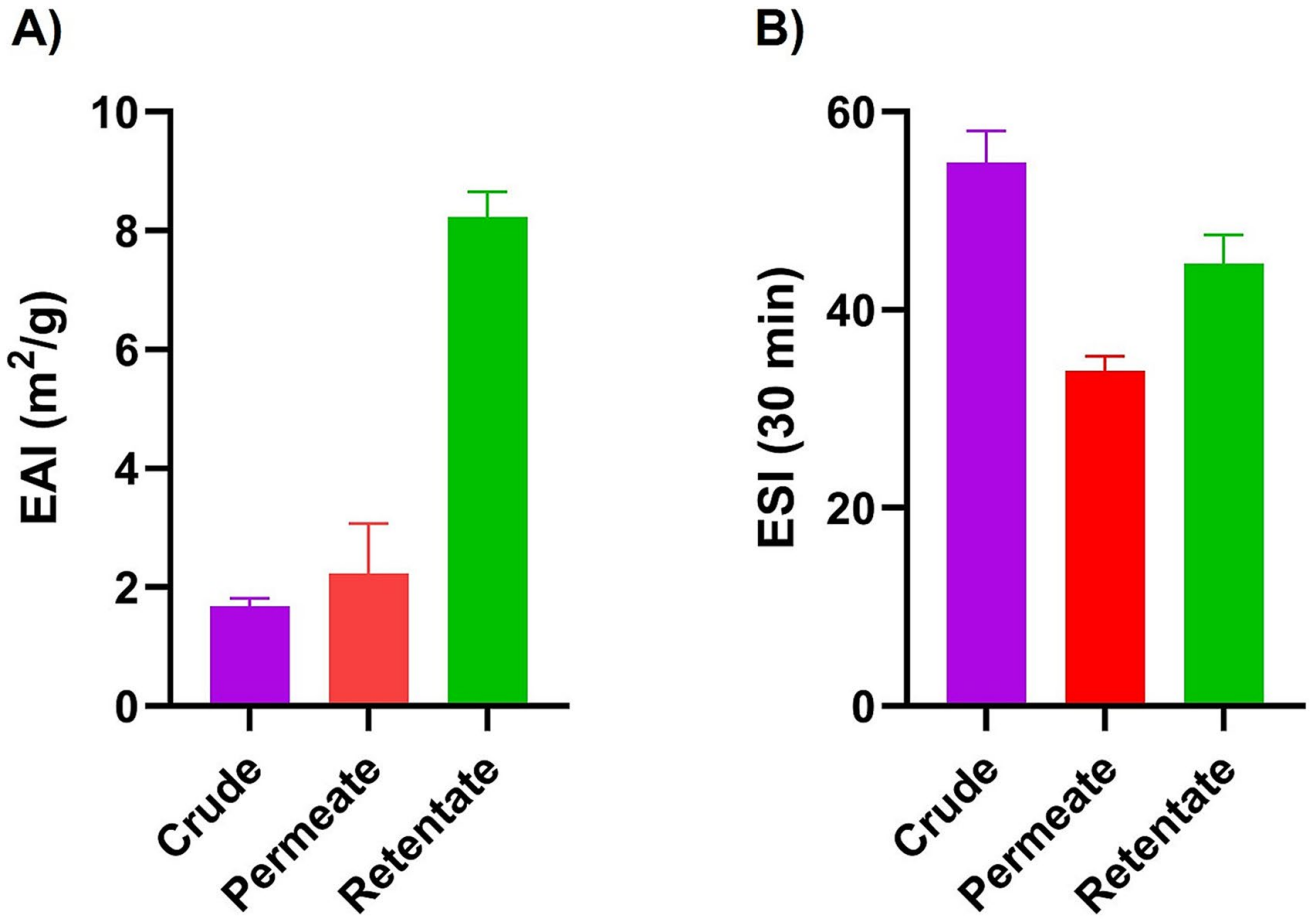
Emulsifying activity index (EAI) **(A)** and emulsifying stability index (ESI) **(B)** of crude, permeate, and retentate (*n* = 3) upon homogenization **(A)** and after 30 min at room temperature **(B)**.

**Figure 4:**
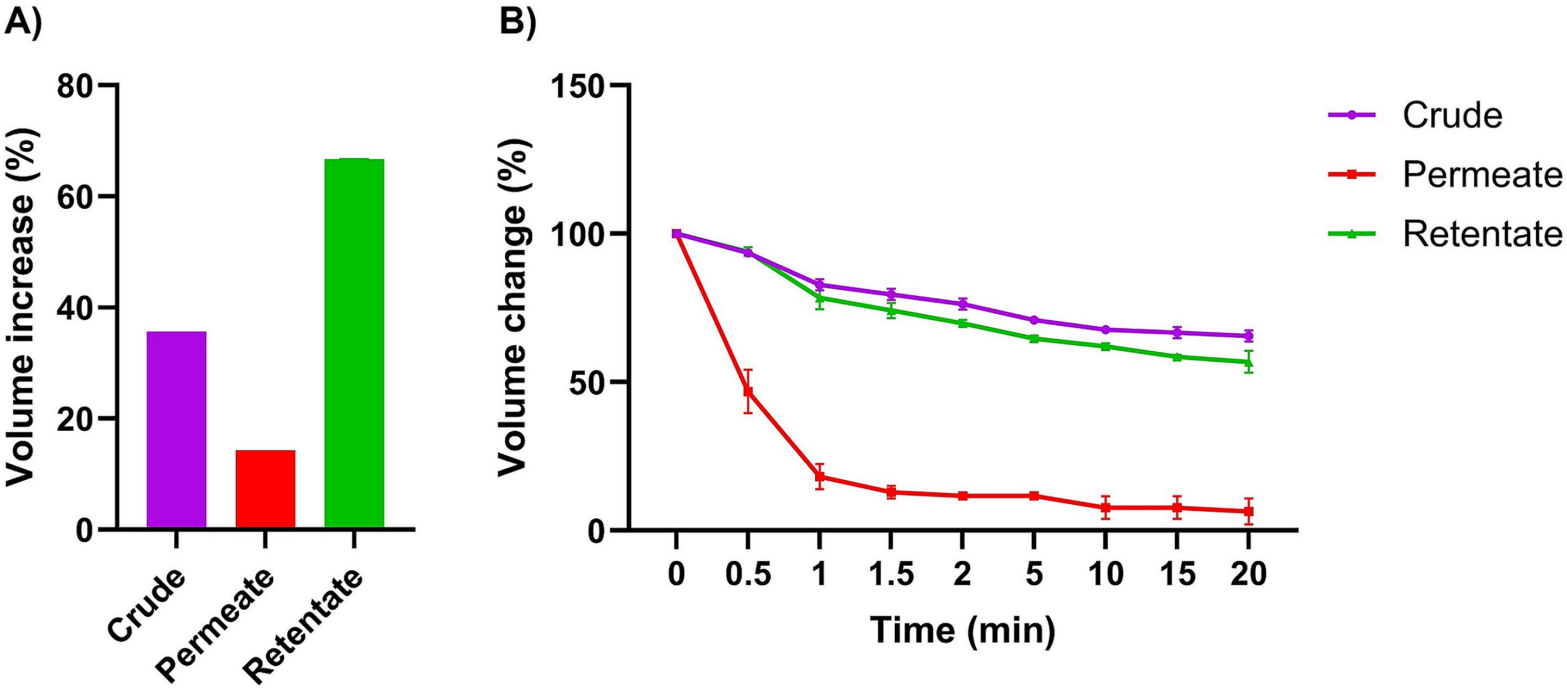
Foaming capacity **(A)** and stability **(B)** of crude, permeate, and retentate (*n* = 3) at 0, 0.5, 1, 1.5, 2, 5-, 10-, 15-, and 20-min following homogenization. Foaming capacity is expressed as volume increase (%) after homogenization, and foaming stability represents % of foam remaining after the respective time intervals.

**Figure 5:**
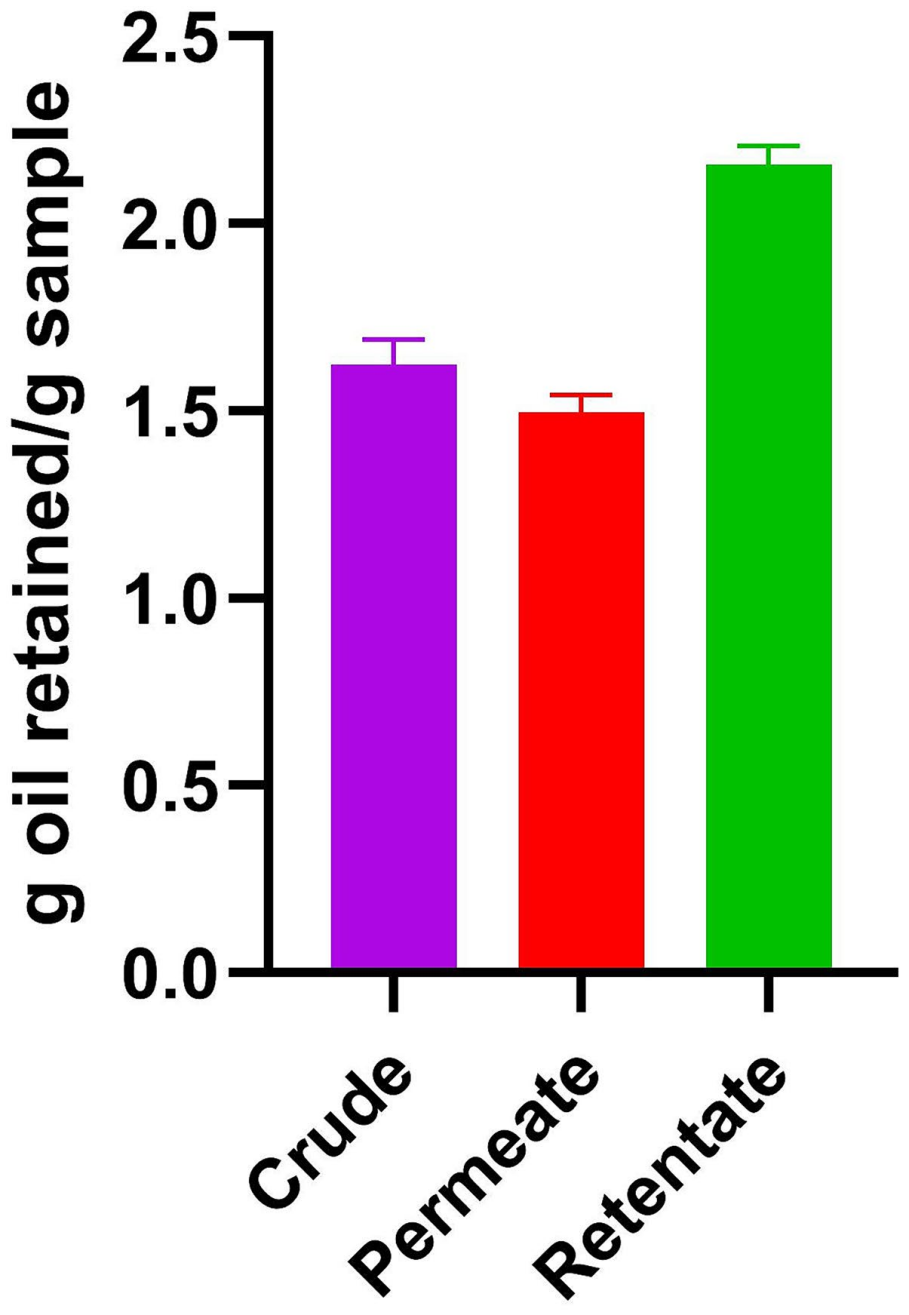
Oil binding capacity of crude, permeate, and retentate (*n* = 3) as expressed in g retained oil/g hydrolysate sample.

The crude, permeate, and retentate were characterized for several techno-functional properties. Of the three samples investigated, the retentate exhibited the most beneficial techno-functional properties, excelling in emulsifying activity (EAI; Figure 3), foaming capacity (Figure 4), and oil binding capacity (Figure 5). Higher molecular weight (MW) fractions, as seen in the retentate sample (Table 3) can form more cohesive interfacial layers around oil droplets, improving emulsifying properties, in agreement with those of high MW protein fractions of quinoa seed proteins (>10 kDa) (44) and Brewers’ spent grain (>14,5 kDa) (14). The permeate, on the other hand, showed the lowest foaming capacity and foaming and emulsifying stability, corresponding to the low MW of the permeate fraction (Table 3). Previous studies reported higher foaming capacity of high MW fractions due to the formation of more stable polymer around the gas bubbles (14) while others reported reduction of foaming capacity and/or stability with increasing MW, depending on the protein source and hydrolysis conditions (e.g., enzyme) (45, 46). In addition to MW, the hydrophobicity of peptides in hydrolysate fractions can significantly influence their techno-functional properties (13, 47) implying that the enzymatic hydrolysis and fractionation in this study may have altered the balance between hydrophobic and hydrophilic regions within the different fractions. The distinctive amino acid profile and higher fat content of the retentate (Tables 1, 2) may also account for its enhanced ability to form and stabilize emulsions and foams as well as for higher oil binding properties (48). The crude exhibited the highest stability to maintain foam and emulsion over time. The higher AMW and MW distribution of the crude sample (Table 3) may indicate the presence of both high- and low- molecular-weight peptides, which may have allowed more effective interface stabilization (49).

### 3.3 Biological activity characterization

#### 3.3.1 ACE inhibitory activity

**Figure 6:**
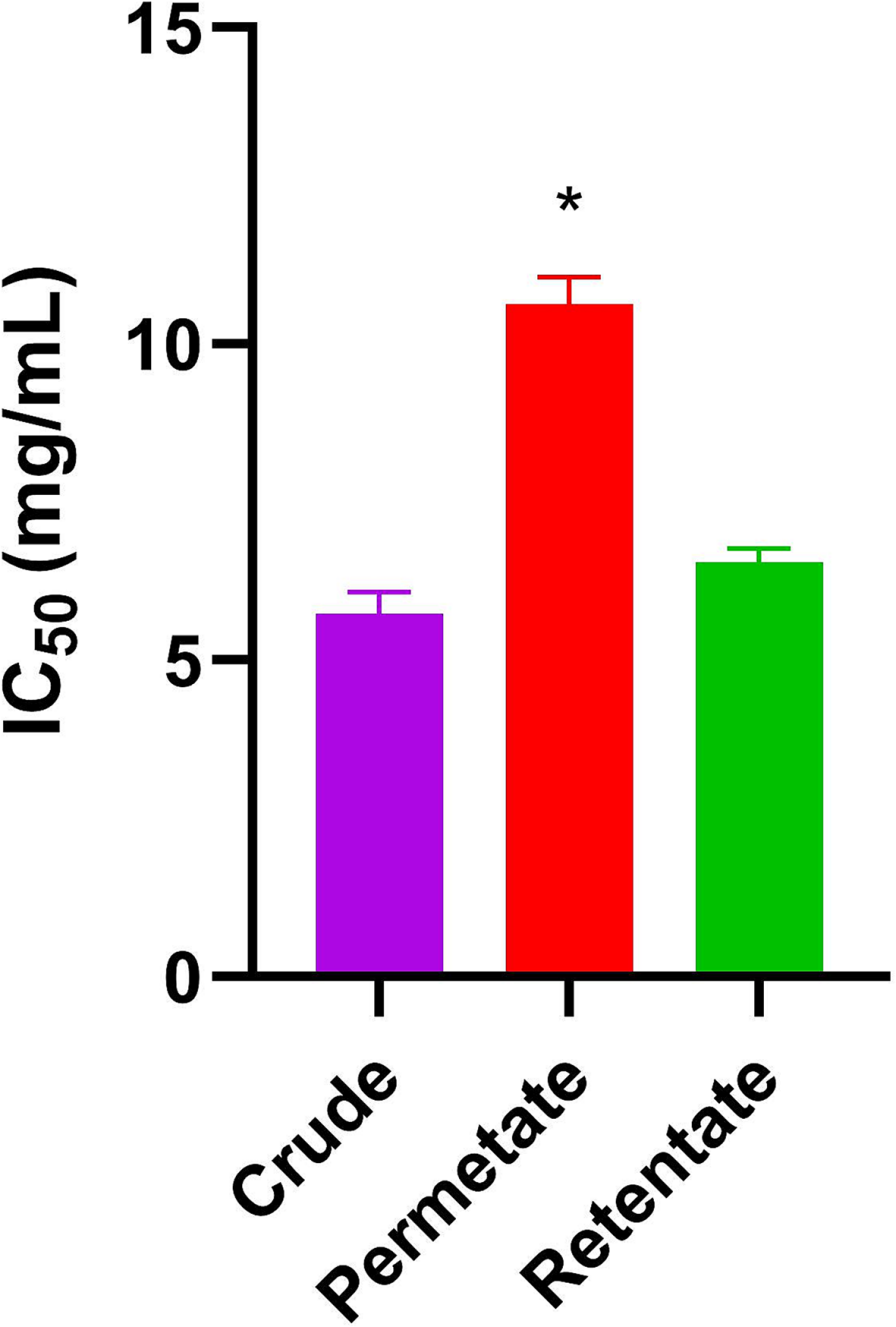
ACE inhibitory activity of crude, permeate, and retentate represented by mean IC_50_ values (mg/mL). The assay was conducted with technical triplicates, and the bars represent the error. *Indicates statistical significant difference between the samples.

The crude, permeate, and retentate samples were analyzed for ACE inhibitory activity (Figure 6). The results are expressed as IC_50_ values, indicating the amount of sample needed to inhibit the biological process by half (50% inhibition of the ACE). Thus, the IC_50_ values provide a measure of the potency of an antagonist compound in pharmacological research. Captopril is a medication used to treat high blood pressure and is a known ACE inhibitor (50). The drug was included in the assay as a control, representing a compound with known and potent ACE inhibitory capacity. The IC_50_ concentration of Captopril in this assay was 0.013 ng/mL. As for the prepared samples, the permeate was the least active, with an IC_50_ of 10.63 mg/mL, followed by retentate (6.56 mg/mL) and crude (5.74 mg/mL). These results differ from the values reported in previous research. A study by Valcarcel (12) reflects extracts from sea bass and sea bream frames that reached IC_50_ values close to 1 mg/mL (12). Differences in methodology and composition of hydrolysates may partially explain this discrepancy. The low IC_50_ values may be due to the synergetic/cumulative effect of various active peptides present in each hydrolysate or aqueous extract, highlighting the need for further identification and characterization of peptides (51). Despite these differences, antihypertensive peptides obtained from seafood have been extensively reviewed elsewhere, demonstrating that the recovery of these bioactive compounds is an efficient way of using seafood by-products (52).

#### 3.3.2 Cellular antioxidant activity

**Figure 7:**
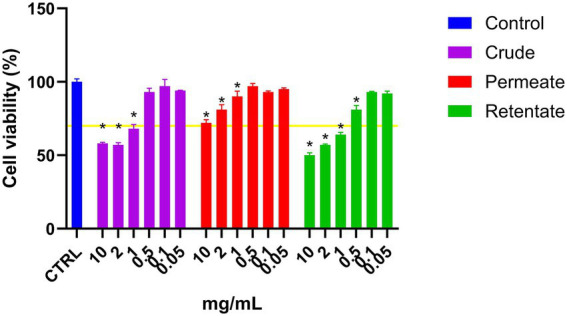
Cell viability of liver cells (Hep G2) treated with crude, permeate, and retentate samples. *Significant differences (*p* < 0.05) with respect to the control (*n* = 3).

**Figure 8:**
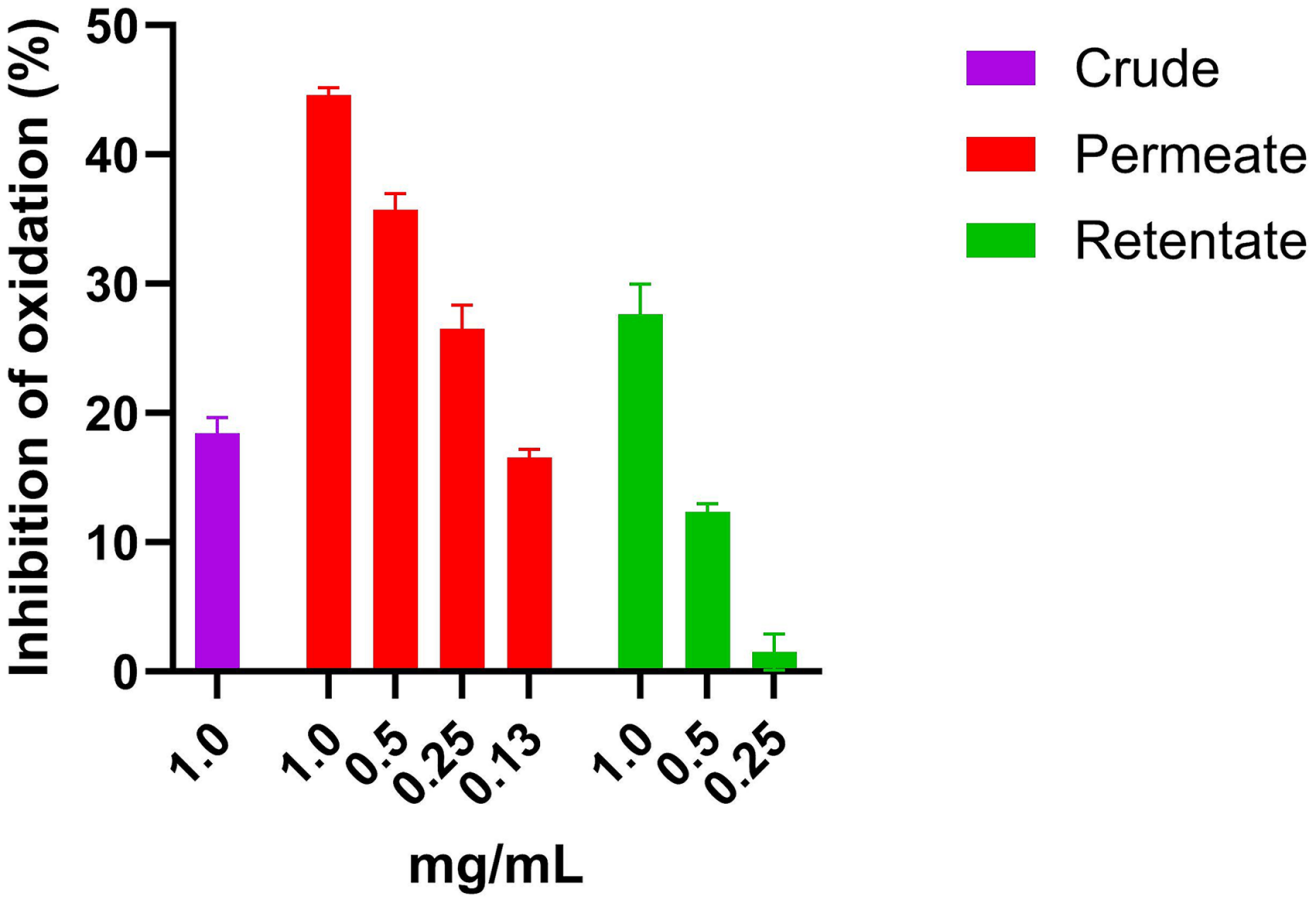
CAA activity of crude, permeate, and retentate samples on Hep G2 cells. The results are shown as mean values with standard deviation (*n* = 3).

The crude, permeate, and retentate were analyzed for CAA on Hep G2 cells. Prior to the CAA, a cell viability assay was performed to determine the highest sample concentrations which did not have a negative effect on cell viability (Figure 7). The cell viability was calculated as percent viability with respect to the control, and for each hydrolysate, the highest concentration that does not affect cell viability was selected for the assay. If cell viability is <70%, the sample is considered to have cytotoxic potential (24). As shown in Figure 7, for both the crude and the retentate, some cytotoxic potential was observed at 1, 2, and 10 mg/mL. No cytotoxic effect was observed for any samples at concentrations 0.5 mg/mL and lower. For the permeate, none of the assayed concentrations presented cytotoxic potential. The results of CAA are commonly presented by IC_50_, this is however not possible as the highest tested concentration that allows cell survival did not inhibit the oxidation above 50%, meaning that the IC_50_ would be an estimated value (Figure 8). Therefore, the results are presented as the inhibition of oxidation, as a function of concentration. Quercetin is included in the assay as a control compound with a known antioxidant capacity, exhibiting an IC_50_ of approximately 5.7 μg/mL. The highest CAA was obtained in the permeate sample, reaching about 45% oxidation inhibition at the highest tested concentration (1 mg/mL). This was expected, as hydrolysates with molecular weights above 3 kDa previously have shown low CAA (53, 54). Molecular weight is a determinant factor for cell absorption. Additionally, factors such as the solubility or polarity of the wide variety of compounds present in hydrolysates contribute to their unique permeability, thereby enabling their bioactivity (54). Dose–response can be observed, as the inhibition reduces with reduced sample concentration. The retentate sample also displays some CAA, with around 27% inhibition at the highest tested concentration (1 mg/mL). The crude sample only showed inhibition at the highest tested concentration (1 mg/mL), reaching only 18% of inhibition.

#### 3.3.3 Anti-inflammatory capacity

**Figure 9:**
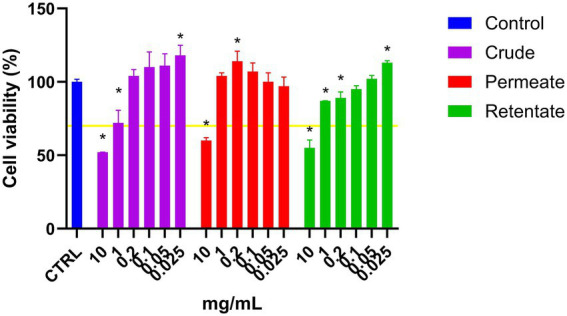
Cell viability of RAW 264.7 cells treated with crude, permeate, and retentate samples in an MTT assay. The results are shown as % cell survival, adjusted to the growth control (*n* = 3). The yellow line represents 70% cell survival, which is considered a cut-off for cytotoxic potential of a sample. *Significant differences (*p* < 0.05) of the hydrolysates with respect to the control.

The crude, permeate, and retentate were analyzed for anti-inflammatory capacity. Prior to the anti-inflammatory assay, the general viability of RAW 264.7 cells treated with samples was assessed. The cell viability was determined by MTT assay (Figure 9), considering a sample to have cytotoxic potential if cell viability is lower than 70% (55). As can be observed in Figure 9, cell survival was above 70% for all samples tested at 1 mg/mL and below. Cell survival increased (above 100%) for several concentrations, indicating a growth effect on the cells. At 10 mg/mL, all samples reduced cell survival below 70%, indicating cytotoxicity at high concentrations. For each sample, the highest concentration that maintained cell viability above 70%, along with the two next lower concentrations, was selected for the anti-inflammatory assay. Therefore, the crude, permeate, and retentate samples were further tested at 1, 0.4, and 0.2 mg/mL in the anti-inflammatory assay.

**Figure 10:**
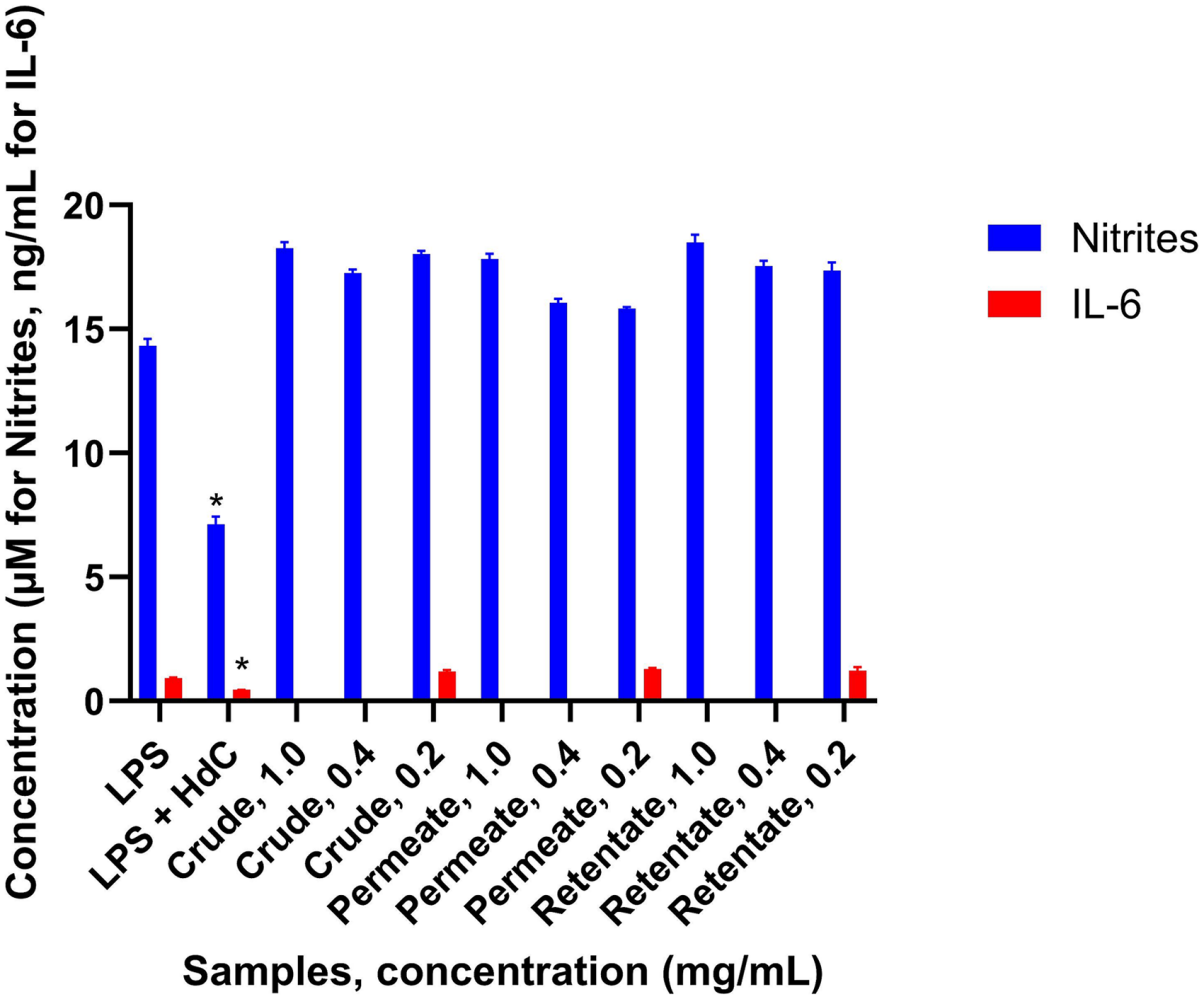
Production of inflammatory factors by the RAW 264.7 cells treated with crude, permeate, and retentate samples in the anti-inflammatory assay (*n* = 3). *Significant differences (*p* < 0.05) in respect to LPS.

The anti-inflammatory assay was performed as described in the methodology. Inflammation was induced using LPS, and the effect of the samples on the inflamed cells was assessed. Nitrite levels were measured across all tested concentrations to evaluate the inflammatory response. Additionally, IL-6 levels were specifically measured in the samples treated at 0.2 mg/mL. The sample was considered to have an anti-inflammatory effect when the production of inflammatory factors was statistically significantly lower (T-test *p* < 0.05) with respect to treatment with LPS (Figure 10). Hydrocortisone (HdC) was used as a positive anti-inflammatory control. No anti-inflammatory capacity was observed from the analyzed protein samples. Although some studies have shown anti-inflammatory effects in hydrolysates obtained from sea bass and sea bream, no anti-inflammatory activity has been detected in the present work. While most studies demonstrating this functionality have utilized extracts from specific tissues - typically skin or heads- very few have investigated extracts from a mixture of tissues (56, 57).

#### 3.3.4 Anti-osteoporotic capacity

**Figure 11:**
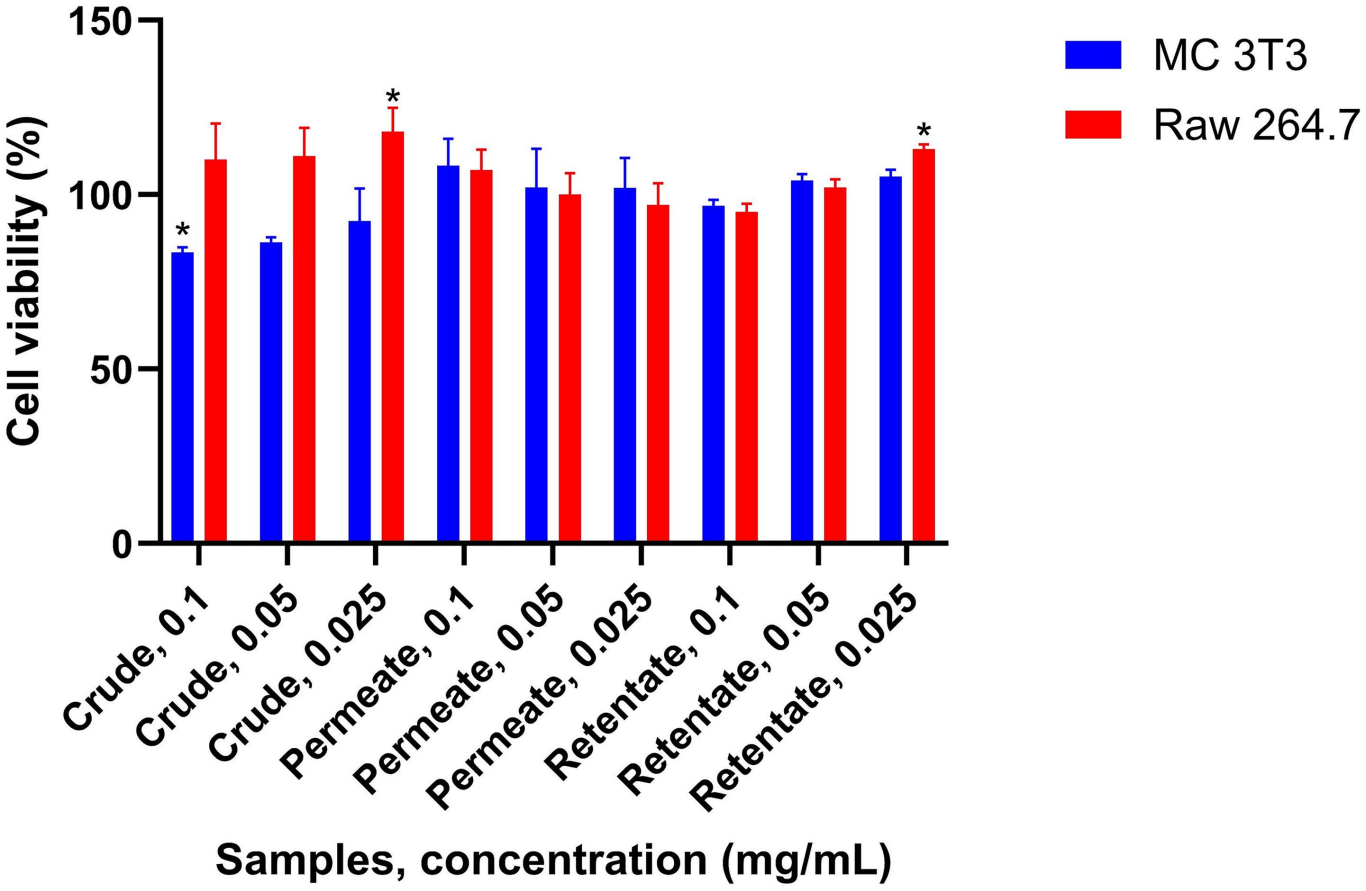
Anti-osteoporotic capacity of the crude, permeate, and retentate measured at the level of cell proliferation of MC 3T3 and RAW 264.7 cells. The effect was evaluated by simultaneous osteoblast proliferation and osteoclast inhibition. The results were expressed as an average relative percentage of proliferation respect to control (*n* = 3). *Significant differences (*p* < 0.05) in respect to control.

There are few known studies that evaluate the anti-osteoporotic capacity of fish hydrolysates. In the case of sea bream and sea bass, this effect has only been demonstrated in collagen hydrolysates derived from sea bass skin (58). The crude, permeate, and retentate were evaluated for their anti-osteoporotic capacity. A sample is considered to have anti-osteoporotic activity if it induces osteoblast growth while simultaneously inhibiting osteoclast growth. The ability of the samples to promote the proliferation and differentiation of osteoblast cells of bone-like matrix on mouse MC3T3-E1 osteoblastic cell subclone 4 (ATCC CRL-2593) was assessed. Also, the capacity to inhibit the preosteoclast proliferation on murine monocyte–macrophage cell line RAW 264.7 (ATCC TIB-71) cells was evaluated. The results of the proliferation study for both cell lines are shown in Figure 11. For all samples, the cell growth of MC 3T3 was higher than 80% with respect to the control, indicating no cytotoxic potential of the samples at the assayed concentrations. However, no significant increase in growth was observed for the osteoblasts. The effect of the samples on the cell proliferation of the RAW 264.7 cells was also determined, showing no reduction in viability at the assayed concentrations. Since none of the samples under the assayed conditions exhibit induction of osteoblast formation and inhibition of osteoclast formation simultaneously, none of the samples are considered to have an osteoprotective effect at the level of cell proliferation.

**Figure 12:**
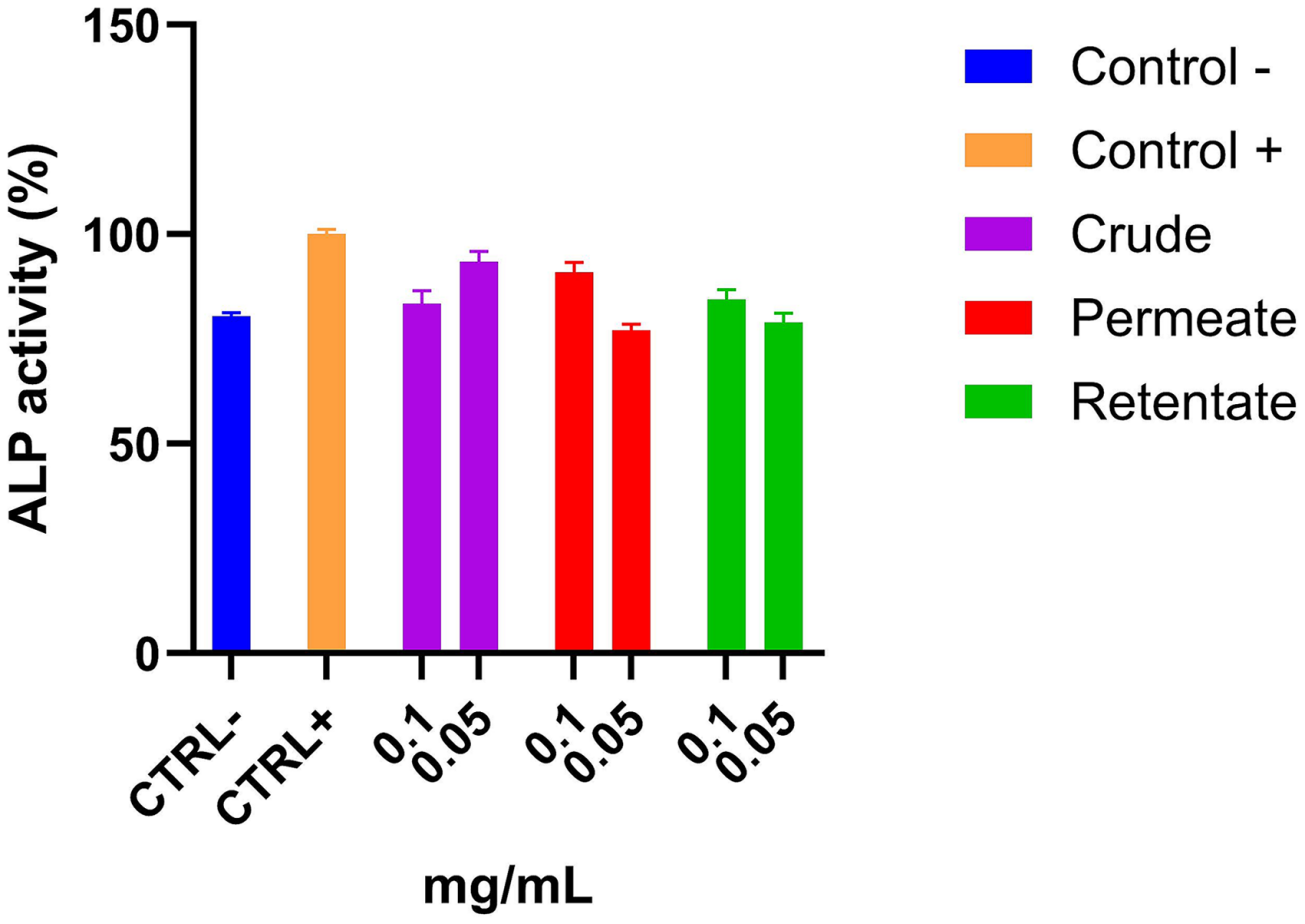
Osteoblast differentiation capacity of the crude, permeate, and retentate, measured by ALP activity of MC 3T3 cells. The results were expressed as the average of the relative percentage of ALP activity with respect to the positive control (*n* = 3).

The crude, permeate, and retentate samples were evaluated in a bone cell differentiation assay. ALP activity, an early marker of osteoblast differentiation and a key indicator of mature osteoblast formation, was quantified to assess the effects of the samples on bone cell development (25). Assays were performed in triplicate with sample concentrations of 0.1 and 0.05 mg/mL. The results are shown as an average of the relative percentage of ALP activity (Figure 12; Supplementary Figure 1). The ALP activity of the positive control refers to the activity under conditions of cell differentiation, while the negative control indicates the activity under normal conditions. If a compound enhances cell differentiation, it should increase the ALP activity of these cells to values higher than the positive control. As expected, the ALP concentration in the negative control was significantly lower than the positive control (Figure 12). None of the samples at the assayed concentrations showed an increase in ALP activity, indicating no anti-osteoporotic capacity.

#### 3.3.5 Hepatoprotective effect against accumulation of fatty acids

**Figure 13:**
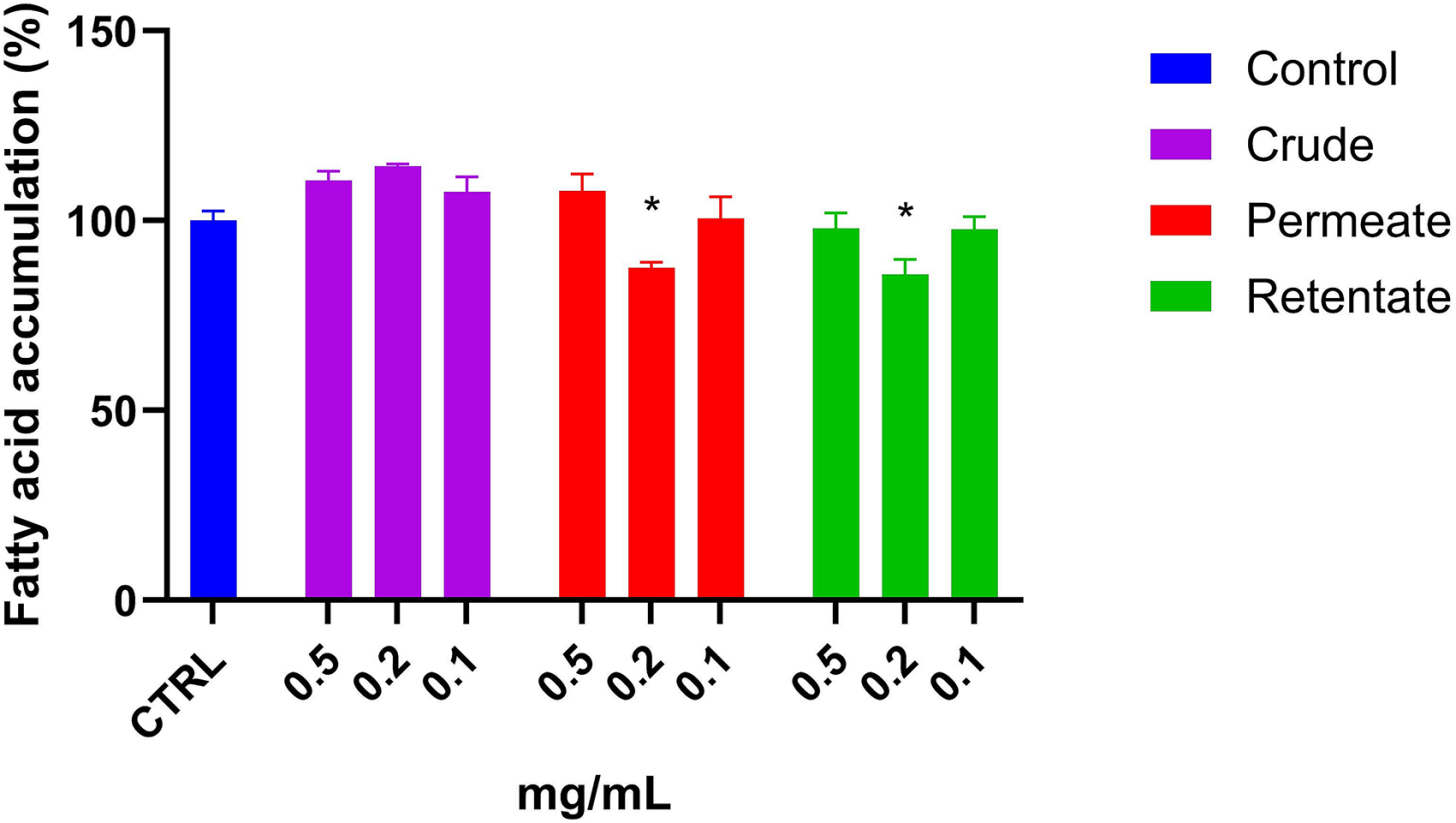
Fatty acid accumulation in Hep G2 cells treated with crude, permeate, and retentate samples. The results were expressed as average of relative percentage of fatty acid respect to control (*n* = 3). *Significant reduction (*p* < 0.05).

To the best of our knowledge, there is no research evaluating the hepatoprotective effect of protein hydrolysates from sea bream and sea bass biomass. The crude, permeate, and retentate samples were assayed for hepatoprotective effect against the accumulation of fatty acids in liver cells. Firstly, a cell viability assay was performed to determine the highest sample concentrations which did not have a negative effect on cell viability (Figure 7). Based on the cell viability results, the hepatoprotective assays were performed in triplicate with sample concentrations of 0.5, 0.2, and 0.1 mg/mL. The results are shown as an average of the relative percentage of inhibition in fatty acid accumulation with respect to control without hydrolysates (Figure 13). The protective effect from the accumulation of fatty acids in hepatic cells is based on the reduction of fatty acid contents in the presence of the tested samples. No effect was observed for the crude sample at the assayed concentrations. Both the permeate and retentate, at 0.2 mg/mL, showed a significant reduction of fatty acid accumulation, as compared to the control. It should be highlighted that the results are based on one biological experiment, and further testing is necessary to confirm these observations.

#### 3.3.6 Cell viability and wound healing activity

**Figure 14:**
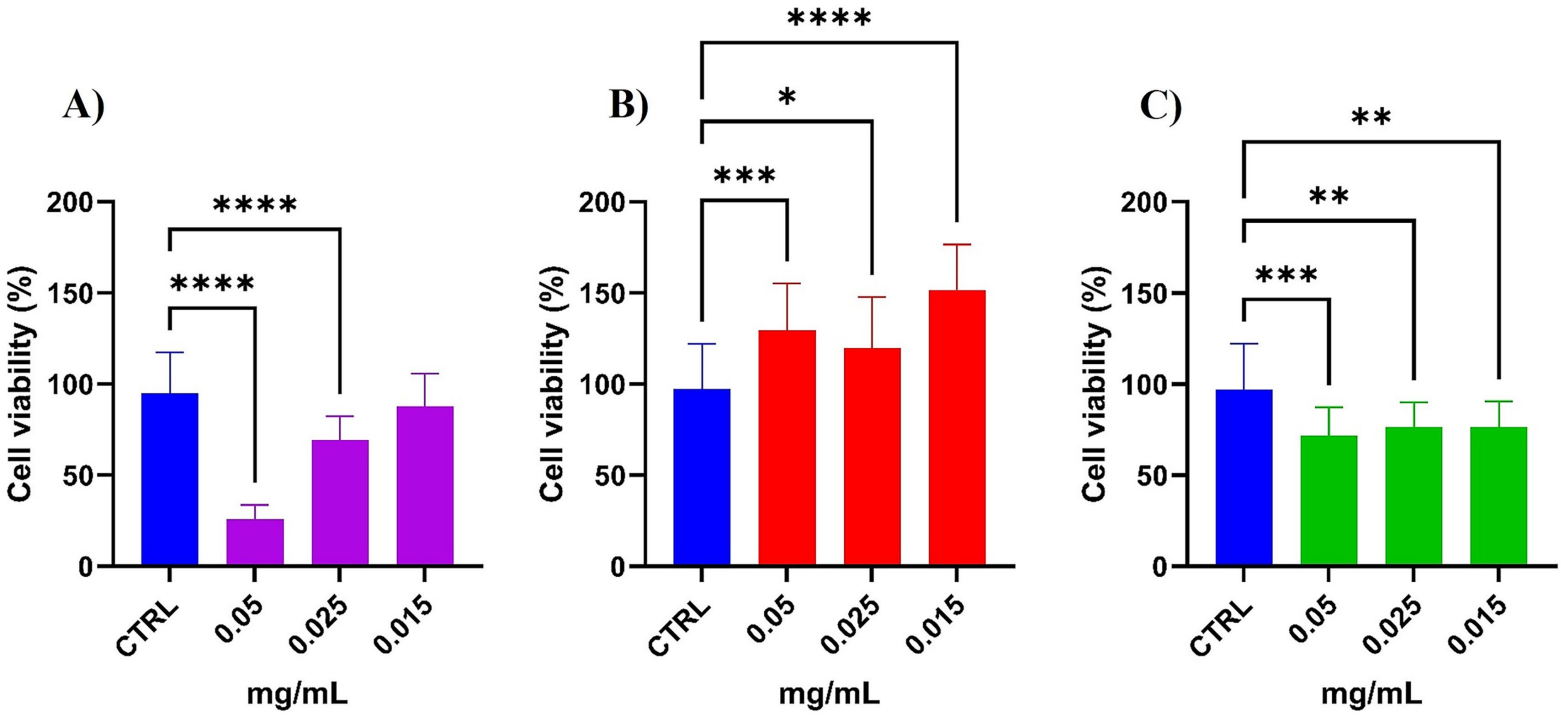
Viability assay on HaCaT cells subjected to **(A)** Crude, **(B)** Permeate, and **(C)** Retentate samples. The results are shown as % cell viability, adjusted to the growth control. Values represented mean ± SD from at least 3 independent experiments. The significant differences are shown as symbols on bars (****p ≤ 0.0001, ***p ≤ 0.001, **p ≤ 0.01, *p ≤ 0.05 vs. CTRL) by one-way analysis of variance (ANOVA) Tukey’s multiple comparison test. Statistical analyses were performed using GraphPad Prism version 10.

The crude, permeate, and retentate samples were evaluated for their effect on the cell viability of HaCaT cells using the MTT assay. As shown in Figure 14, there are significant differences in cell viability compared to the control (no treatment). The crude sample negatively affects cell viability at the highest assayed concentrations (0.05 and 0.025 mg/mL), with cell viability as low as 25 and 65% (Figure 14A). The permeate, on the other hand, increases the cell viability at all tested concentrations, compared to the control, reaching approximately 150% cell viability at 0.015 mg/mL (Figure 14B). The retentate has a negative effect on cell viability, compared to the control, at all tested concentrations (Figure 14C).

**Figure 15:**
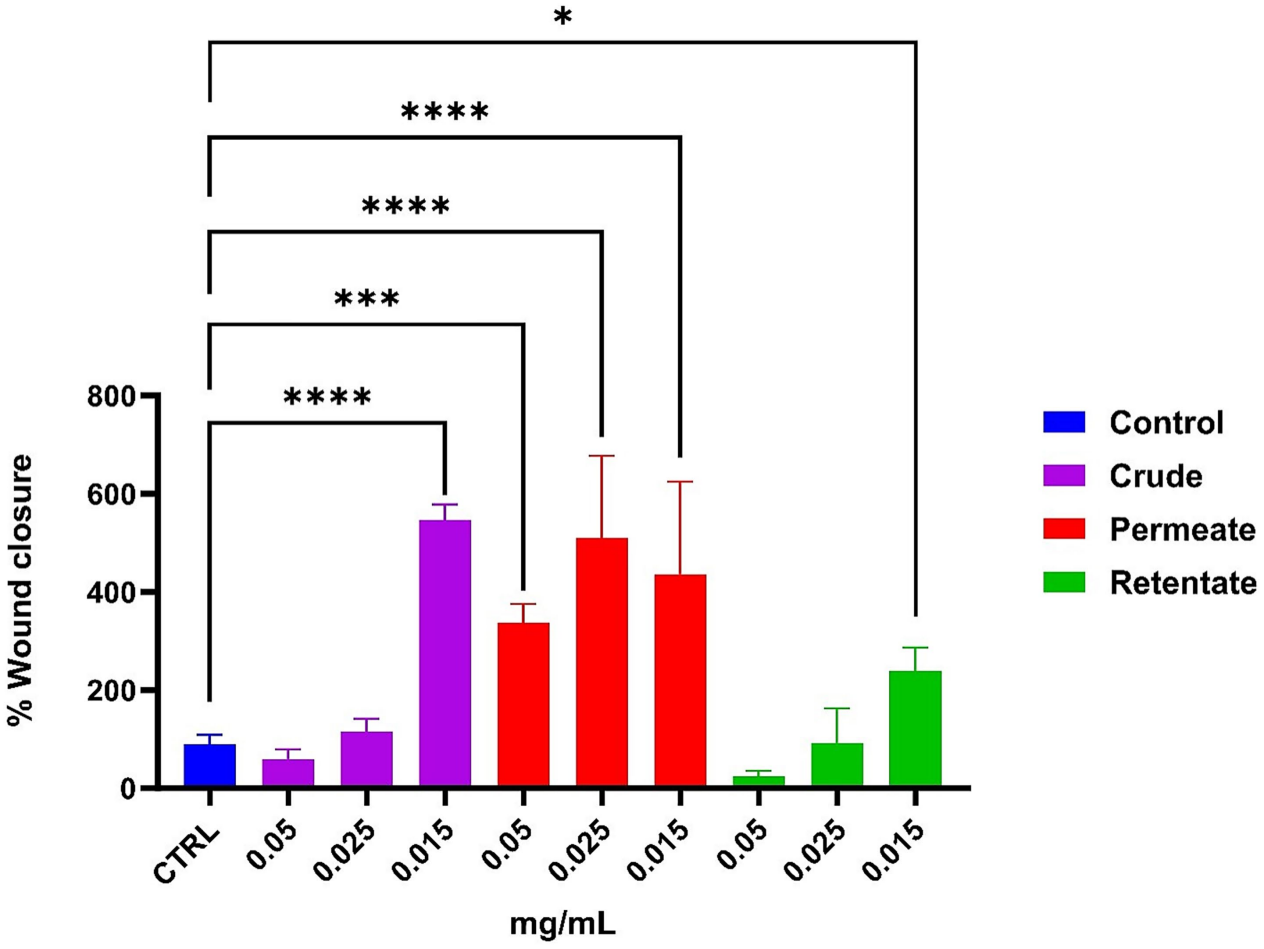
Effect on wound healing, measured by a T-scratch test on human keratinocytes. The effect of the Crude, Permeate, and Retentate is evaluated at different concentrations. Values mean ± SD from at least 3 independent experiments. The significant differences are shown as symbols on bars (****p ≤ 0.0001, ***p ≤ 0.001, *p ≤ 0.05 vs. CTRL) by one-way analysis of variance (ANOVA) Tukey’s multiple comparison test. Statistical analyses were performed using GraphPad Prism version 10.

To assess potential wound healing capacities, the crude, permeate, and retentate samples were evaluated in a T-scratch assay on human keratinocyte cells. Figure 15 shows significant differences in the percentage of wound closure on the HaCaT cells after 24 h of incubation. For the crude, a positive effect is observed at the lowest concentration (0.015 mg/mL), reaching about 550% wound closure (compared to the control). For the permeate, all the concentrations positively affected wound healing capacity, with around 500% at 0.025 mg/mL compared to the control. For the retentate, a positive impact can be observed at the lowest concentration (0.015 mg/mL), reaching about 200%. As documented in the literature, peptide size has been demonstrated to significantly influence wound closure activity (59–61). Images were taken at time 0 (T0) and time 24 h (T24) compared to control (shown in Supplementary Figure 2).

The obtained results showed that cells treated with the crude extract at the lowest concentration (0.015 mg/mL) exhibited no significant difference in cell viability compared to the negative control, while presenting a wound healing capacity. In cells treated with the permeate, a correlation was observed between cell viability and the t-scratch assay results. Indeed, all tested concentrations of the permeate extract had a positive effect on both cell viability and wound healing activity. Cells treated with the retentate showed decreased viability at all tested concentrations. However, the t-scratch assay revealed a contrasting trend: the lowest concentration of the retentate extract promoted wound healing. This discrepancy may be attributed to the molecular weight-based separation, which appears to influence cellular responses affecting cell viability.

## 4 Conclusion

Recovery, utilization, and valorization of aquaculture side streams play a crucial role in increasing the sustainability of this industry. Rather than being treated as waste, these residual biomasses can become valuable raw materials for developing and producing bioactive peptides, functional ingredients, and other high-value products. The approach not only contributes to reducing the environmental footprint but also supports innovation and circularity in the marine-based economy, opening new avenues in food, nutraceutical, cosmetic, and pharmaceutical applications. This study explored the valorization of dehydrated side streams from farmed sea bream and sea bass through enzymatic hydrolysis and subsequent fractionation. Dehydration was employed to stabilize the biomass and reduce its volume and weight, facilitating more efficient storage and transportation. The primary objective was to comprehensively evaluate the chemical composition, techno-functional attributes, and biological activities of the resulting hydrolysate and its two fractions: the permeate and retentate. All samples exhibited high protein content, exceeding 80% as determined by the Kjeldahl method (N x 6.25), underscoring their potential as protein-rich ingredients. Size exclusion chromatography analysis revealed that the retentate possessed the highest average molecular weight, followed by the crude and the permeate, aligning with the expectations based on the fractionation process. Colorimetric analysis indicated that the permeate was notably lighter (higher L* value), distinguishing it visually from the other fractions. Techno-functional assessments demonstrated that the retentate exhibited superior properties, including the highest emulsifying activity, foaming capacity, and oil-binding capacity. These characteristics suggest strong potential for the retentate’s application in food formulation requiring functional protein ingredients. The hydrolysate and its fractions were broadly assessed for biological activity, including assaying for antihypertensive, antioxidant, anti-inflammatory, anti-osteoporotic, hepatoprotective, and wound healing activities. The samples did not exhibit anti-inflammatory activity or anti-osteoporotic capacity. Some activity was observed in the hepatoprotective assay (reduced fatty acid accumulation), the cellular antioxidant assay, and the antihypertensive assay. However, the T-scratch assay on HaCaT keratinocyte cells revealed particularly promising wound-healing potential in the permeate fraction, showing great activity at all tested concentrations. Overall, this study highlights the feasibility and value of enzymatic hydrolysis and membrane fractionation as strategies for converting aquaculture side streams into high-value functional and bioactive ingredients. These findings support the development of sustainable bioprocessing approaches for the production of specialized ingredients suitable for use in food, nutraceutical and potentially cosmeceutical applications.

## Data Availability

The original contributions presented in the study are included in the article/Supplementary material, further inquiries can be directed to the corresponding author.
