# Supplementary material for: Valorization of aquaculture side streams from sea bream and sea bass by enzymatic hydrolysis and fractionation: chemical and biological insights
Source: Front Nutr. 2025 Sep 26;12:1663294. doi: 10.3389/fnut.2025.1663294 (PMC12512169; doi:10.3389/fnut.2025.1663294)
Supplement: Supplementary file 1 [file Data_Sheet_1.PDF]

Supplementary Material

1 Osteoblast differentiation

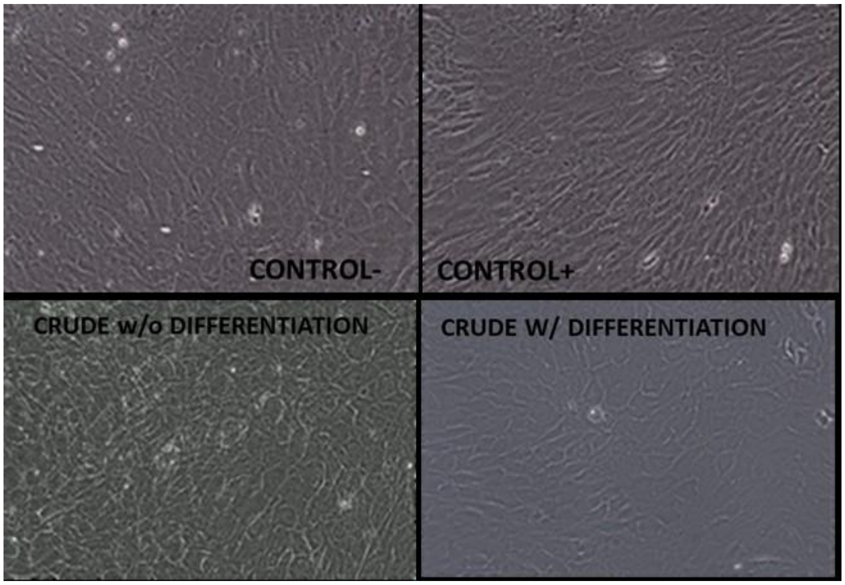

Supplementary Figure 1: Microscopic view of osteoblast differentiation activity of cells treated with crude sample (on the right side without differentiation treatment, and on the left side with differentiation treatment) compared to the positive and negative control.

2 Wound healing assay on HaCaT cell line

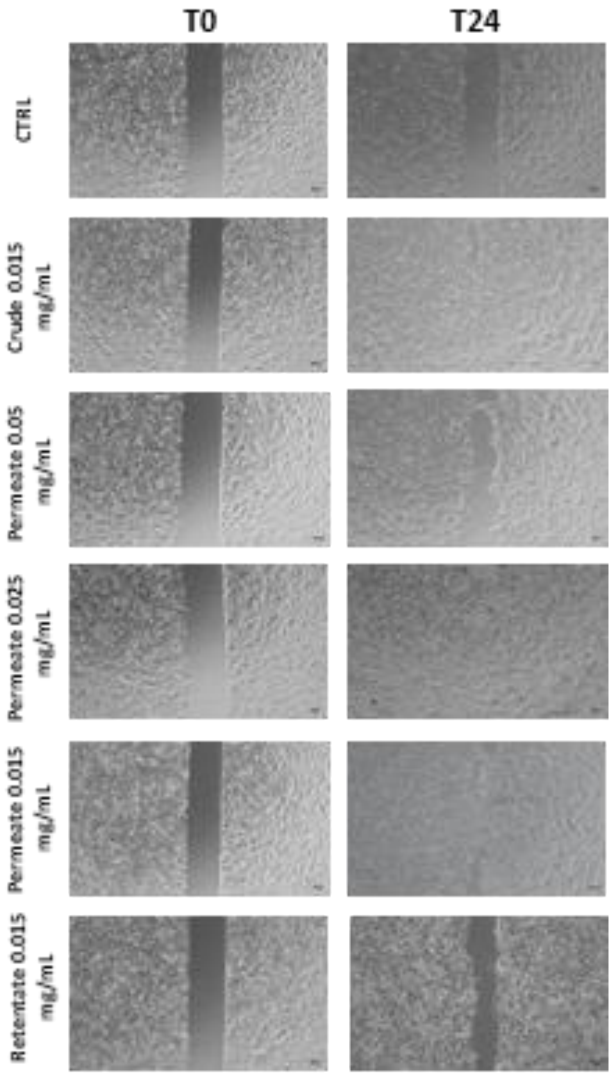

Supplementary Figure 2. Representative images of the effect of Crude, Permeate and Retentate samples and CTRL on HaCaT cell line using the wound healing assay at time zero (T0) and after 24 hours (T24).
